# Supplementary figures and images for: Regulation of positive and negative selection and TCR signaling during thymic T cell development by capicua
Source: eLife. 2021 Dec 13;10:e71769. doi: 10.7554/eLife.71769 (PMC8700290; doi:10.7554/eLife.71769)

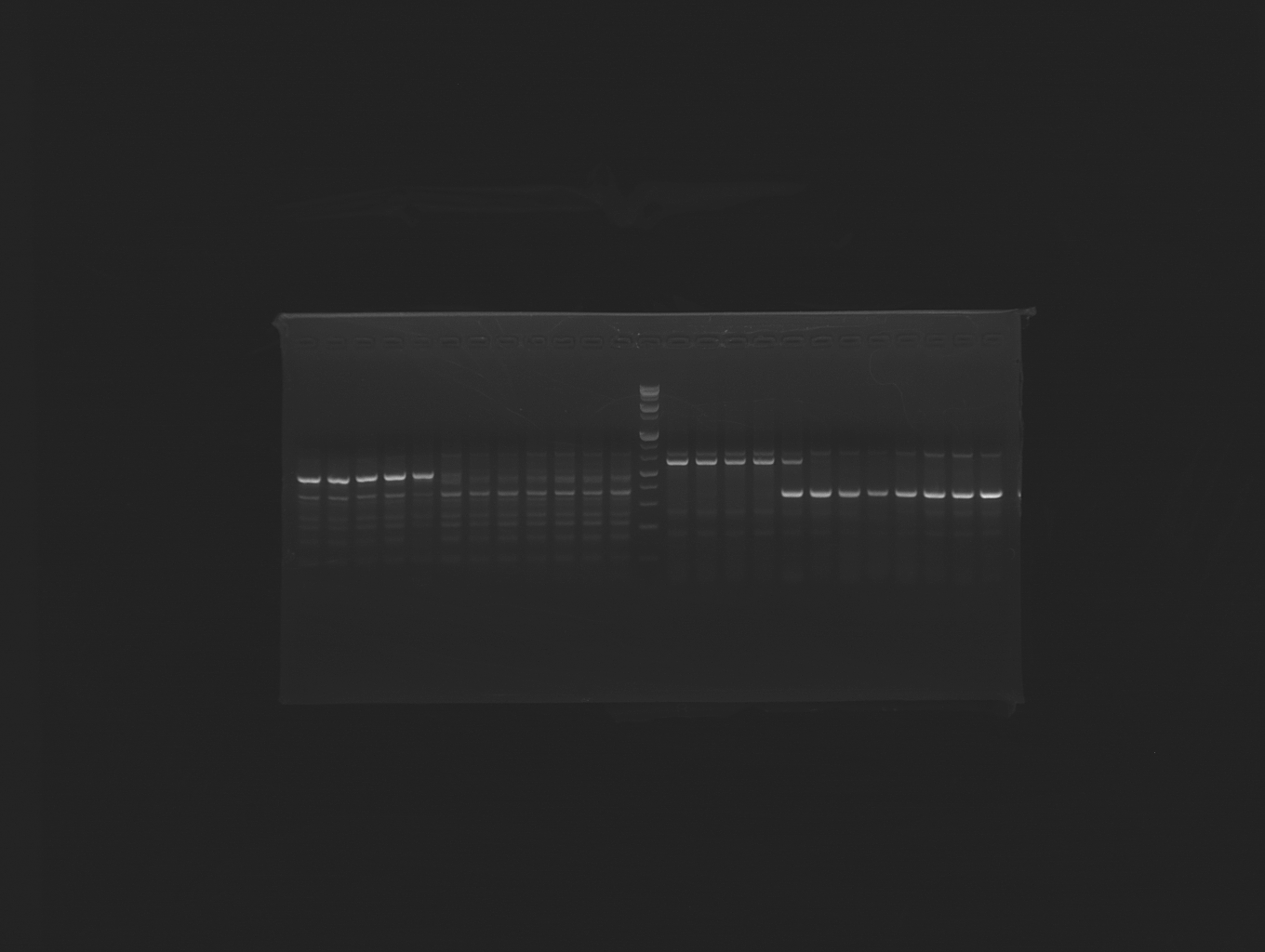

Supplement: Figure 2—source data 1. [file elife-71769-fig2-data1.zip › F2_pcr gel image_original file.tif]

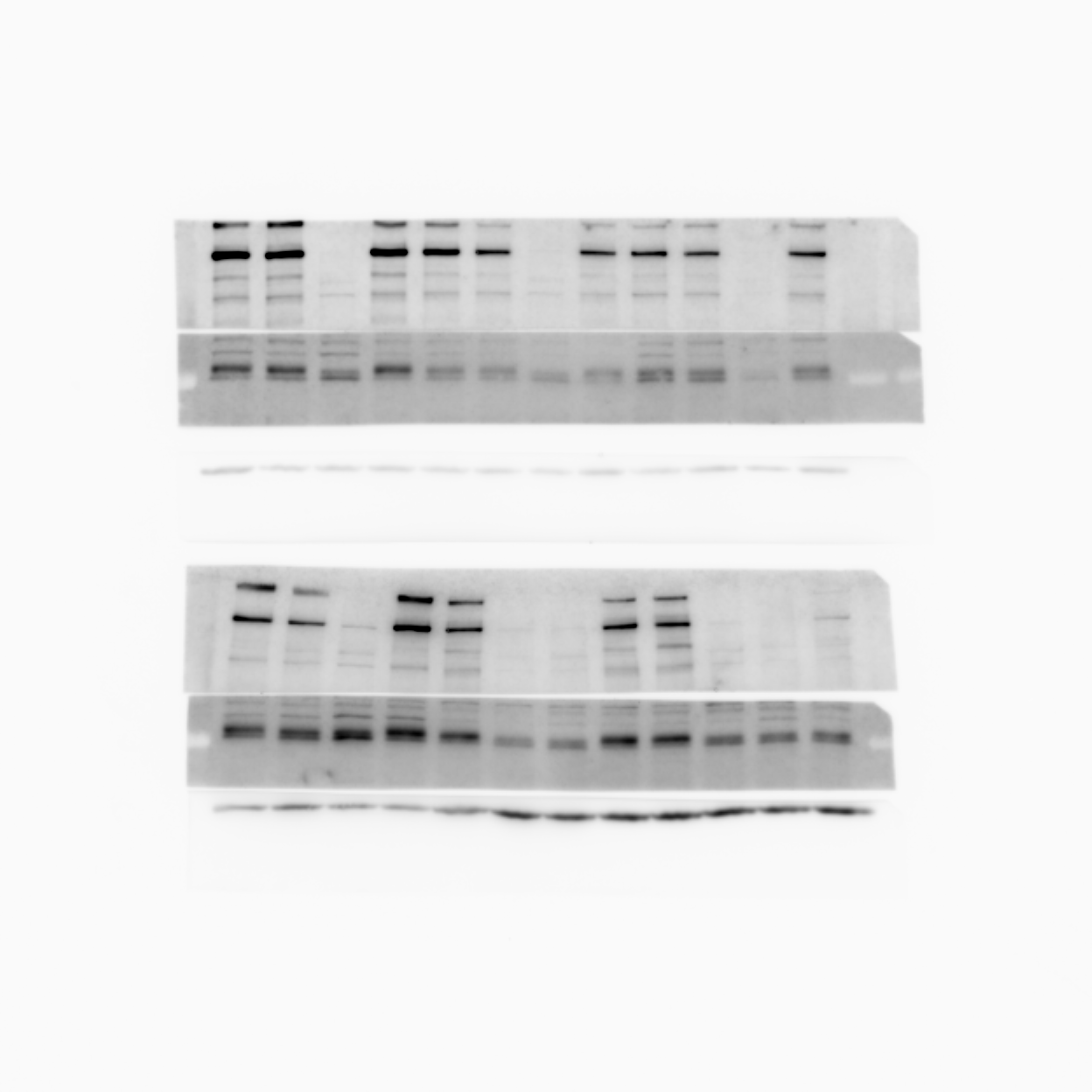

Supplement: Figure 2—source data 1. [file elife-71769-fig2-data1.zip › F2_western blot_original file_1.tif]

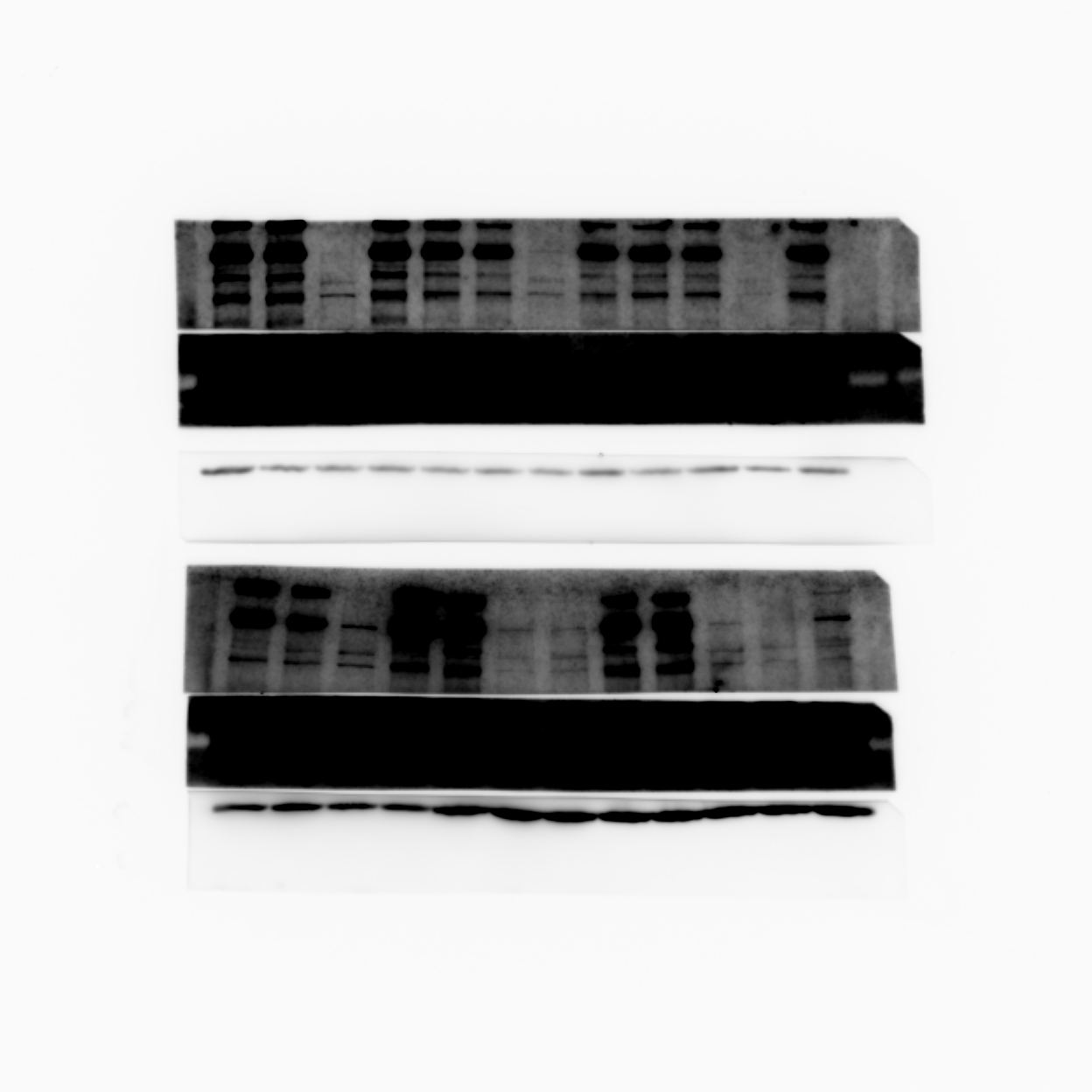

Supplement: Figure 2—source data 1. [file elife-71769-fig2-data1.zip › F2_western blot_original file_2.tif]

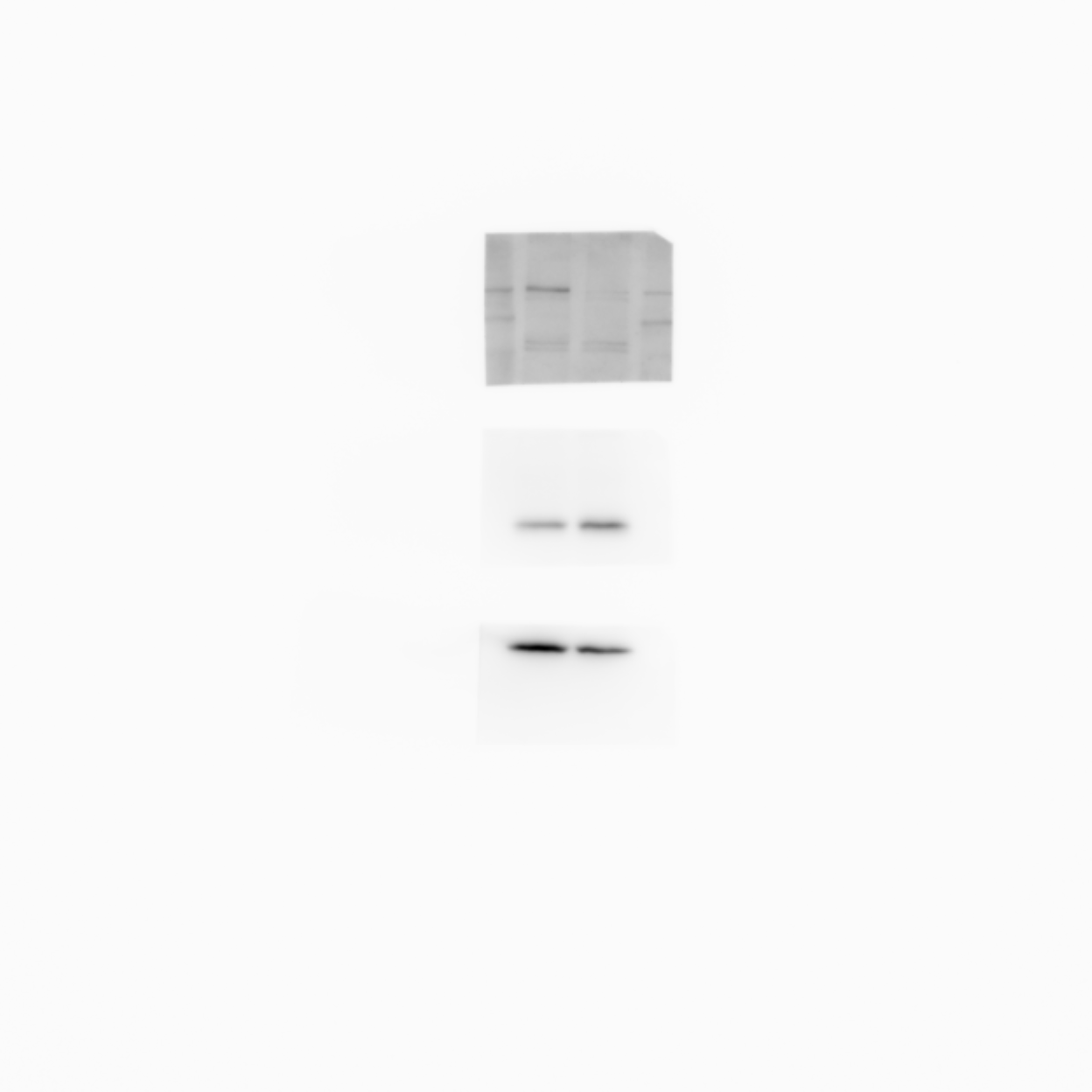

Supplement: Figure 2—source data 1. [file elife-71769-fig2-data1.zip › F2_western blot_original file_3.tif]

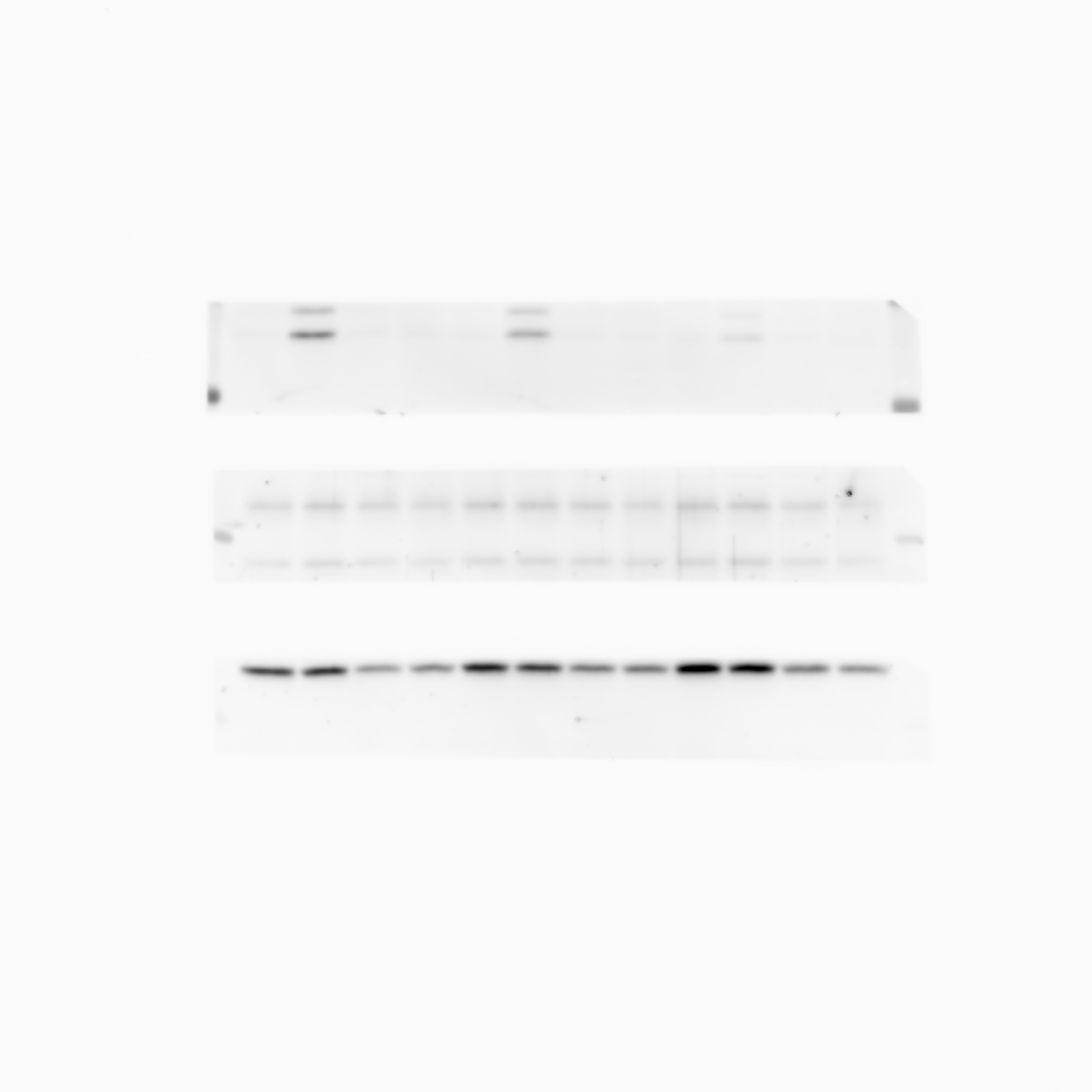

Supplement: Figure 5—source data 2. [file elife-71769-fig5-data2.zip › F5_western blot_original file_1.tif]

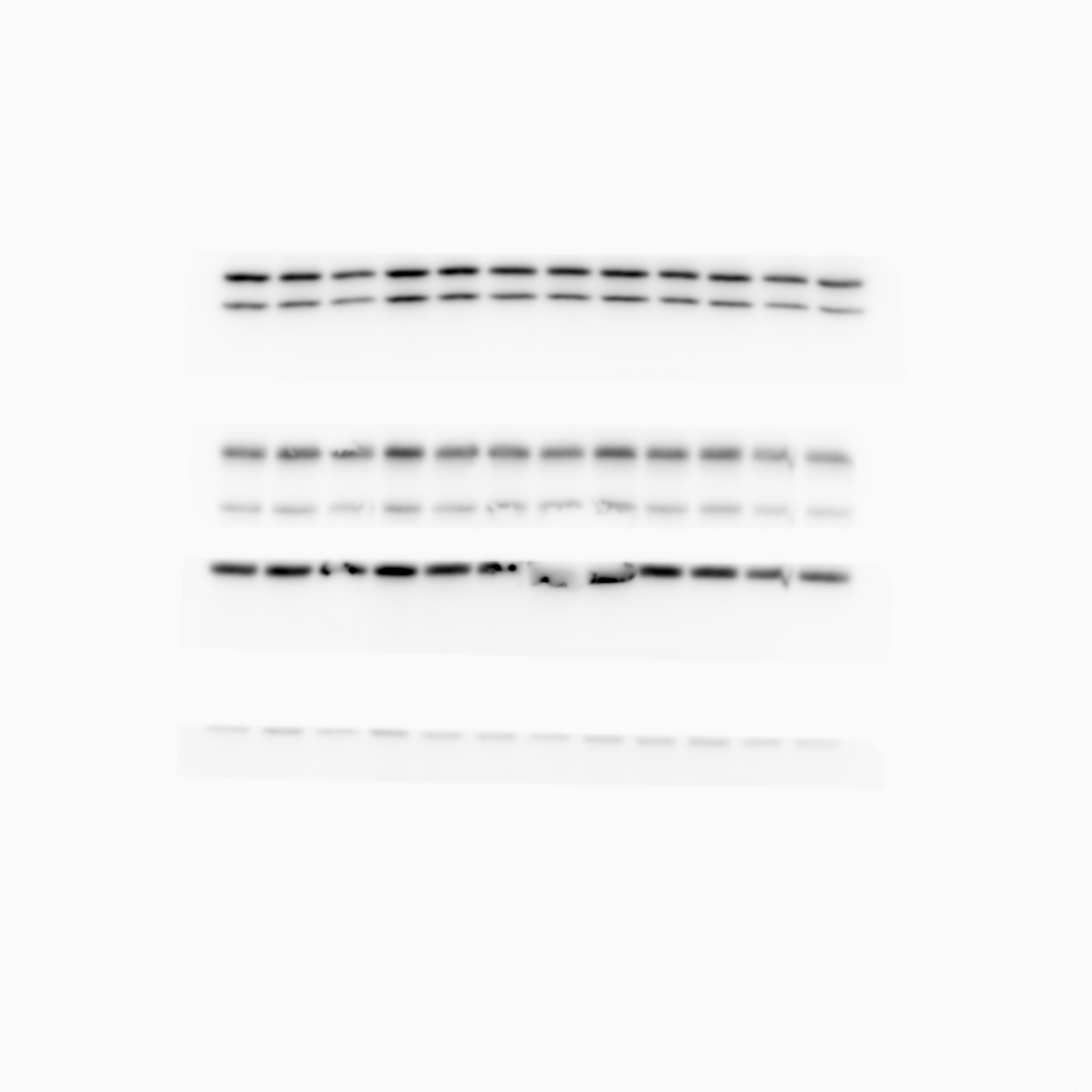

Supplement: Figure 5—source data 2. [file elife-71769-fig5-data2.zip › F5_western blot_original file_2.tif]

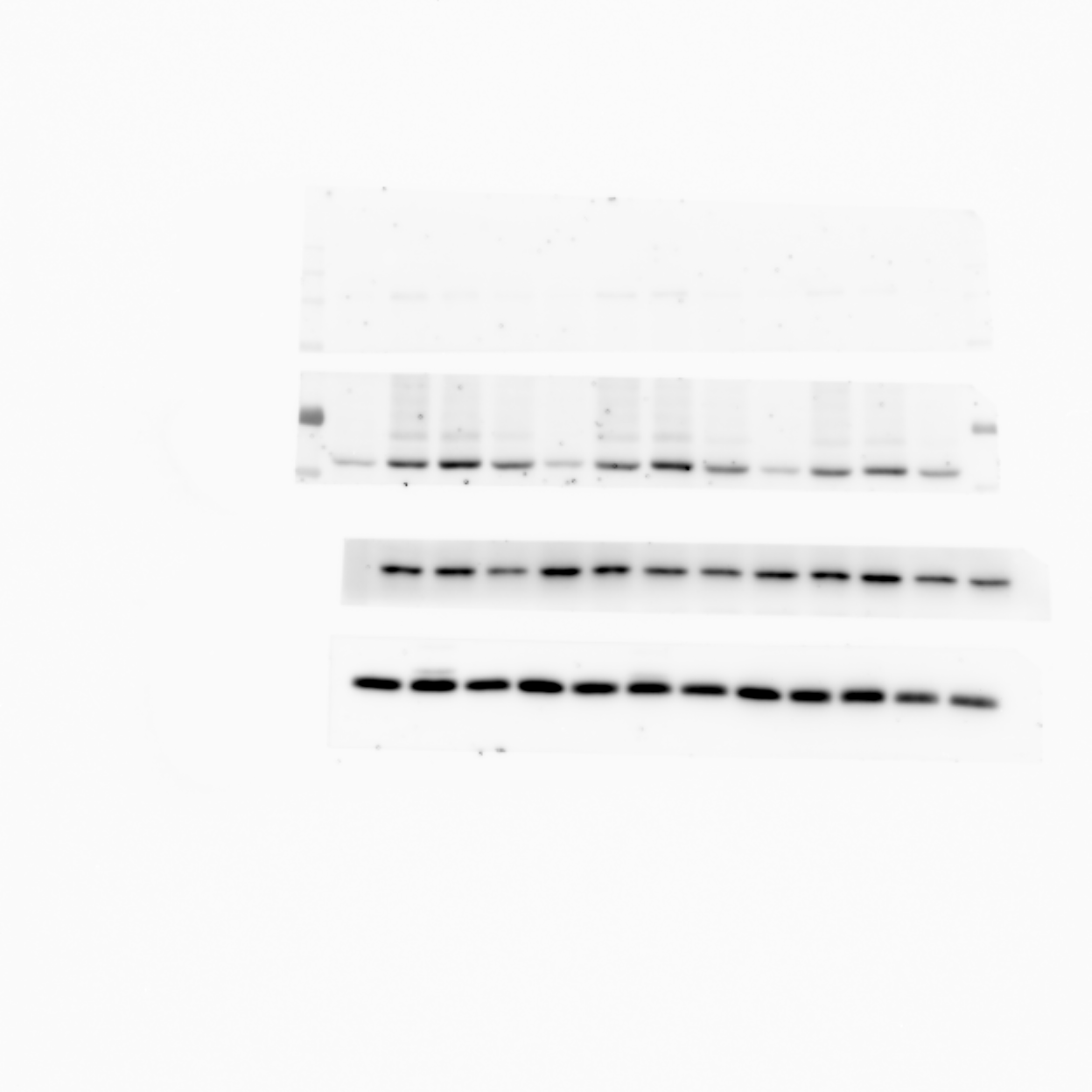

Supplement: Figure 5—source data 2. [file elife-71769-fig5-data2.zip › F5_western blot_original file_3.tif]

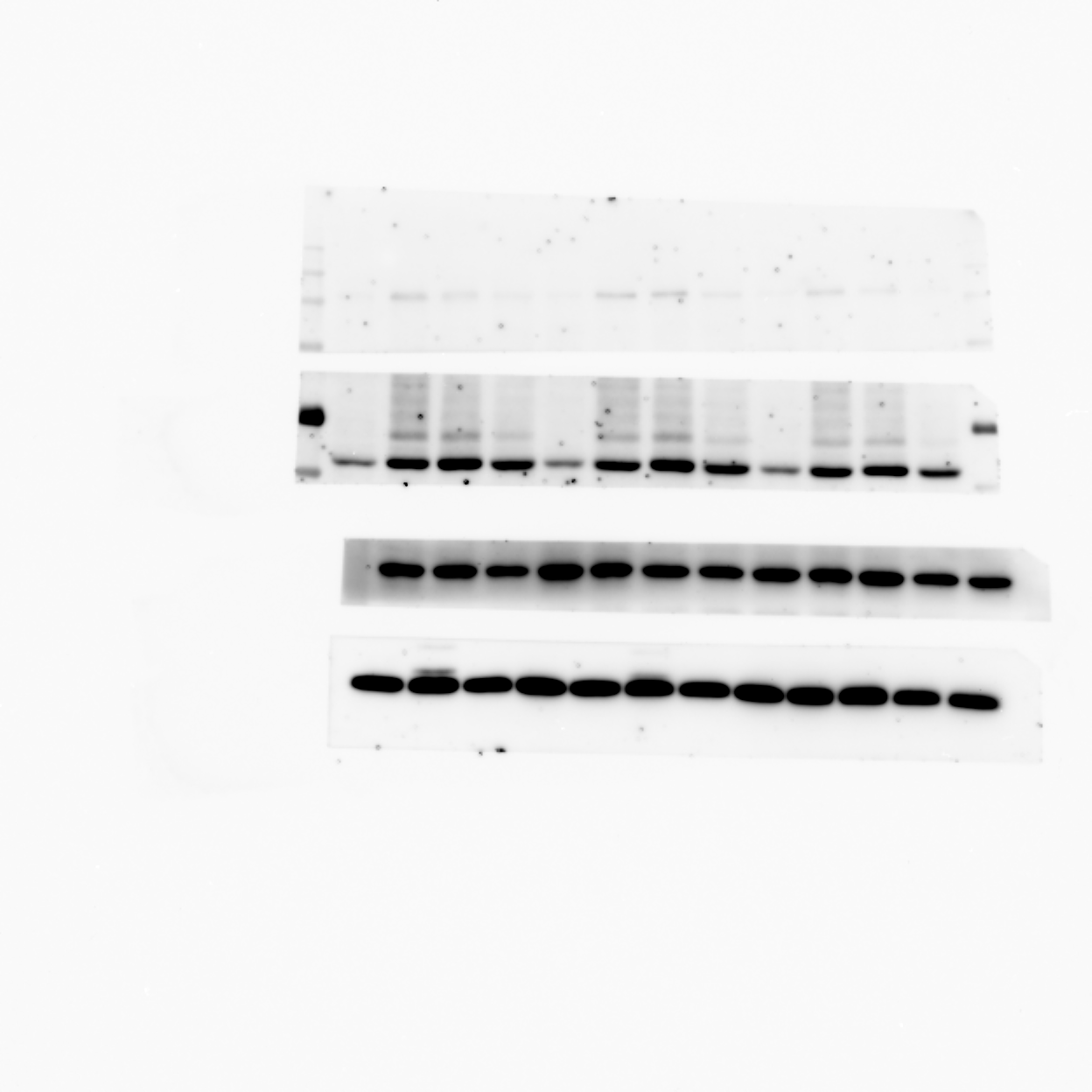

Supplement: Figure 5—source data 2. [file elife-71769-fig5-data2.zip › F5_western blot_original file_4.tif]

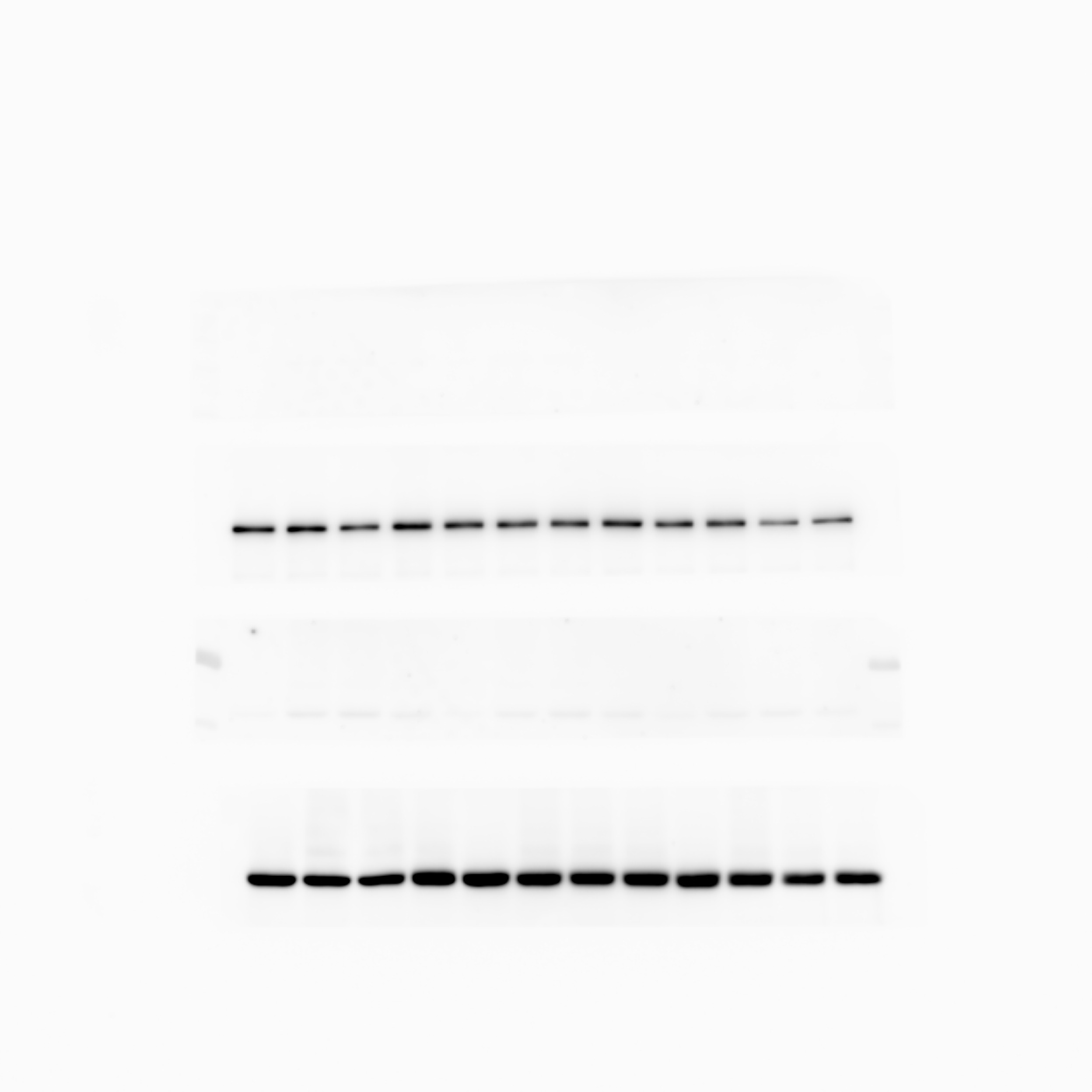

Supplement: Figure 5—source data 2. [file elife-71769-fig5-data2.zip › F5_western blot_original file_5.tif]
