# Supplementary material for: Regulation of positive and negative selection and TCR signaling during thymic T cell development by capicua
Source: eLife. 2021 Dec 13;10:e71769. doi: 10.7554/eLife.71769 (PMC8700290; doi:10.7554/eLife.71769)
Supplement: Supplementary file 1. [file elife-71769-supp1.docx]

| **Table S1. Differentially expressed genes (DEGs) in *Cic*-deficient DP thymocytes** | | | | | |
| --- | --- | --- | --- | --- | --- |
| Gene_ID | Gene_Symbol | **KO/WT.fc** | KO/WT.raw.pval | KO/WT.bh.pval | |
| 18612 | Etv4 | **231.247** | **1.675E-63** | | **2.682E-59** |
| 24066 | Spry4 | **39.348** | **2.365E-24** | | **1.262E-20** |
| 104156 | Etv5 | **21.748** | **9.148E-46** | | **7.322E-42** |
| 28240 | Trpm2 | **18.631** | **3.214E-05** | | **5.254E-03** |
| 17260 | Mef2c | **18.250** | **4.837E-16** | | **7.040E-13** |
| 100134861 | Gm44502 | **17.490** | **9.483E-13** | | **1.168E-09** |
| 216082 | Gm4798 | **14.136** | **2.127E-10** | | **1.480E-07** |
| 115488204 | Smim32 | **12.326** | **4.438E-09** | | **2.153E-06** |
| 66785 | 4933433G08Rik | **12.168** | **1.803E-07** | | **5.890E-05** |
| 102633937 | Gm31639 | **10.733** | **1.253E-09** | | **7.430E-07** |
| 214239 | Ccdc9b | **10.485** | **4.345E-07** | | **1.364E-04** |
| 14009 | Etv1 | **10.148** | **4.035E-16** | | **6.459E-13** |
| 214791 | Sertad4 | **10.069** | **1.486E-17** | | **2.975E-14** |
| 108169205 | LOC108169205 | **9.615** | **2.595E-08** | | **1.123E-05** |
| 13390 | Dlx1 | **9.411** | **1.766E-10** | | **1.285E-07** |
| 16071 | Igkc | **8.900** | **6.811E-04** | | 6.945E-02 |
| 115486208 | LOC115486208 | **7.544** | **8.095E-04** | | 7.950E-02 |
| 115487281 | LOC115487281 | **7.377** | **8.131E-03** | | 3.708E-01 |
| 56644 | Clec7a | **6.804** | **4.540E-05** | | **7.197E-03** |
| 17381 | Mmp12 | **6.580** | **4.092E-08** | | **1.590E-05** |
| 105244595 | Gm40185 | **6.506** | **1.597E-08** | | **7.520E-06** |
| 102633038 | Gm30959 | **6.181** | **2.152E-04** | | **2.817E-02** |
| 16517 | Kcnj16 | **6.123** | **1.150E-03** | | 1.017E-01 |
| 12978 | Csf1r | **5.942** | **2.407E-03** | | 1.751E-01 |
| 12262 | C1qc | **5.831** | **4.420E-06** | | **9.434E-04** |
| 269610 | Chd5 | **5.624** | **1.941E-03** | | 1.501E-01 |
| 66011 | Ranbp17 | **5.470** | **1.484E-04** | | **2.031E-02** |
| 237625 | Pla2g3 | **5.459** | **2.126E-03** | | 1.605E-01 |
| 228731 | Nkx2-4 | **5.312** | **1.291E-03** | | 1.096E-01 |
| 677321 | Gm9712 | **5.285** | **1.536E-02** | | 5.554E-01 |
| 319520 | Dusp4 | **4.869** | **2.347E-20** | | **7.515E-17** |
| 16409 | Itgam | **4.864** | **4.975E-18** | | **1.138E-14** |
| 100316663 | Mir669k | **4.820** | **9.158E-03** | | 4.017E-01 |
| 271849 | Shc4 | **4.762** | **1.354E-06** | | **3.675E-04** |
| 16971 | Lrp1 | **4.761** | **1.483E-10** | | **1.131E-07** |
| 66395 | Ahnak | **4.590** | **2.725E-17** | | **4.848E-14** |
| 432572 | Specc1 | **4.562** | **4.614E-03** | | 2.610E-01 |
| 100416030 | Gm17879 | **4.546** | **1.012E-03** | | 9.310E-02 |
| 115488120 | LOC115488120 | **4.415** | **2.166E-03** | | 1.622E-01 |
| 102637682 | Gm26682 | **4.375** | **5.217E-05** | | **8.108E-03** |
| 258571 | Olfr1033 | **4.282** | **3.346E-04** | | **3.998E-02** |
| 79221 | Hdac9 | **4.260** | **8.312E-07** | | **2.420E-04** |
| 115489725 | LOC115489725 | **4.247** | **6.149E-03** | | 3.165E-01 |
| 67603 | Dusp6 | **4.234** | **5.354E-22** | | **2.143E-18** |
| 57269 | Olfr1507 | **4.233** | **9.944E-04** | | 9.255E-02 |
| 108168851 | Gm38237 | **4.231** | **6.789E-04** | | 6.945E-02 |
| 14369 | Fzd7 | **4.228** | **3.292E-07** | | **1.054E-04** |
| 319616 | Fzd10os | **4.215** | **4.333E-04** | | **4.851E-02** |
| 117167 | Steap4 | **4.164** | **4.031E-06** | | **8.881E-04** |
| 115487985 | LOC115487985 | **4.100** | **2.569E-02** | | 7.082E-01 |
| 13040 | Ctss | **4.074** | **1.125E-05** | | **2.218E-03** |
| 100038577 | Gm10790 | **4.069** | **1.743E-02** | | 5.913E-01 |
| 106522 | Pkdcc | **4.062** | **1.846E-03** | | 1.449E-01 |
| 228911 | Tshz2 | **4.044** | **1.413E-04** | | **1.951E-02** |
| 105242938 | Gm39010 | **4.016** | **3.485E-02** | | 8.090E-01 |
| 23831 | Car14 | **4.000** | **3.948E-03** | | 2.403E-01 |
| 18627 | Per2 | **3.998** | **8.699E-07** | | **2.487E-04** |
| 71398 | 5430427O19Rik | **3.976** | **3.317E-02** | | 7.907E-01 |
| 17386 | Mmp13 | **3.952** | **5.542E-03** | | 2.927E-01 |
| 102465194 | Mir6374 | **3.895** | **1.850E-02** | | 6.140E-01 |
| 433365 | Teddm1b | **3.870** | **1.204E-03** | | 1.054E-01 |
| 75202 | Spaca6 | **3.813** | **2.860E-02** | | 7.442E-01 |
| 114715 | Spred1 | **3.795** | **6.548E-10** | | **4.032E-07** |
| 100503338 | Gm14636 | **3.752** | **2.023E-04** | | **2.676E-02** |
| 105246219 | Gm41532 | **3.717** | **3.071E-02** | | 7.621E-01 |
| 102640671 | Gm36685 | **3.684** | **1.657E-02** | | 5.833E-01 |
| 59036 | Dact1 | **3.666** | **3.114E-08** | | **1.278E-05** |
| 223927 | Gtsf2 | **3.637** | **1.319E-02** | | 5.064E-01 |
| 115490302 | LOC115490302 | **3.636** | **1.304E-02** | | 5.020E-01 |
| 12862 | Cox6a2 | **3.612** | **1.277E-03** | | 1.094E-01 |
| 72748 | Hdhd3 | **3.601** | **1.044E-02** | | 4.295E-01 |
| 56461 | Kcnip3 | **3.581** | **2.019E-06** | | **5.131E-04** |
| 105244320 | Gm39954 | **3.577** | **2.034E-02** | | 6.390E-01 |
| 102644 | Oaf | **3.571** | **2.557E-04** | | **3.249E-02** |
| 432800 | Gm5454 | **3.569** | **4.508E-02** | | 9.056E-01 |
| 207393 | Elfn2 | **3.550** | **2.363E-02** | | 6.821E-01 |
| 100503495 | A930016O22Rik | **3.506** | **3.055E-02** | | 7.617E-01 |
| 115489534 | LOC115489534 | **3.483** | **2.039E-03** | | 1.547E-01 |
| 216190 | Appl2 | **3.478** | **7.844E-03** | | 3.655E-01 |
| 216197 | Ckap4 | **3.434** | **1.489E-02** | | 5.418E-01 |
| 105246679 | Gm37053 | **3.416** | **2.449E-06** | | **6.031E-04** |
| 115489127 | LOC115489127 | **3.409** | **4.741E-02** | | 9.222E-01 |
| 230612 | Slc5a9 | **3.395** | **3.438E-09** | | **1.720E-06** |
| 17533 | Mrc1 | **3.391** | **2.500E-03** | | 1.772E-01 |
| 19341 | Rab4a | **3.381** | **1.463E-02** | | 5.365E-01 |
| 17067 | Ly6c1 | **3.372** | **7.657E-05** | | **1.114E-02** |
| 102643244 | Gm16252 | **3.362** | **7.738E-03** | | 3.647E-01 |
| 320706 | Soga1 | **3.354** | **2.892E-04** | | **3.553E-02** |
| 93708 | Pcdhgc5 | **3.325** | **9.674E-03** | | 4.108E-01 |
| 105246945 | Gm42139 | **3.313** | **4.165E-03** | | 2.460E-01 |
| 102631635 | Gm29927 | **3.307** | **3.721E-03** | | 2.320E-01 |
| 231014 | 9330182L06Rik | **3.303** | **9.538E-04** | | 8.982E-02 |
| 105244068 | Gm39746 | **3.302** | **3.045E-05** | | **5.102E-03** |
| 320051 | Exph5 | **3.294** | **2.145E-02** | | 6.572E-01 |
| 115488309 | LOC115488309 | **3.283** | **3.171E-02** | | 7.741E-01 |
| 102640071 | Gm36227 | **3.262** | **4.600E-02** | | 9.111E-01 |
| 14012 | Mpzl2 | **3.253** | **2.473E-03** | | 1.772E-01 |
| 100040500 | Gm2808 | **3.246** | **1.270E-07** | | **4.325E-05** |
| 105244392 | Gm40011 | **3.245** | **4.795E-03** | | 2.665E-01 |
| 102637109 | Gm34004 | **3.237** | **8.677E-03** | | 3.891E-01 |
| 20720 | Serpine2 | **3.235** | **4.480E-05** | | **7.173E-03** |
| 26912 | Gcat | **3.231** | **4.597E-02** | | 9.111E-01 |
| 69565 | 2310015K22Rik | **3.231** | **7.588E-03** | | 3.612E-01 |
| 329152 | Hecw2 | **3.211** | **2.787E-06** | | **6.659E-04** |
| 105243151 | Gm39150 | **3.197** | **7.112E-03** | | 3.482E-01 |
| 14800 | Gria2 | **3.164** | **4.337E-06** | | **9.382E-04** |
| 102640320 | Gm36417 | **3.105** | **2.562E-03** | | 1.783E-01 |
| 100040471 | Gm2792 | **3.091** | **1.451E-06** | | **3.808E-04** |
| 54486 | Hpgds | **3.090** | **3.225E-02** | | 7.788E-01 |
| 20271 | Scn5a | **3.075** | **3.493E-10** | | **2.237E-07** |
| 12443 | Ccnd1 | **3.062** | **2.703E-02** | | 7.271E-01 |
| 320642 | A630066F11Rik | **3.039** | **2.712E-02** | | 7.286E-01 |
| 215303 | Camk1g | **3.027** | **1.750E-05** | | **3.295E-03** |
| 69456 | Commd10 | **3.014** | **5.636E-08** | | **2.050E-05** |
| 23963 | Tenm1 | **3.012** | **4.921E-08** | | **1.832E-05** |
| 69660 | Tmbim1 | **3.010** | **2.573E-02** | | 7.082E-01 |
| 329324 | Syt14 | **3.007** | **2.745E-10** | | **1.831E-07** |
| 65962 | Slc9a3r2 | **2.978** | **7.194E-03** | | 3.490E-01 |
| 277154 | Nynrin | **2.976** | **7.794E-05** | | **1.124E-02** |
| 101631 | Pwwp2b | **2.969** | **5.076E-03** | | 2.802E-01 |
| 12259 | C1qa | **2.966** | **2.465E-03** | | 1.772E-01 |
| 73619 | 1700122E12Rik | **2.964** | **1.641E-02** | | 5.821E-01 |
| 54377 | Cacng4 | **2.957** | **5.039E-03** | | 2.791E-01 |
| 11676 | Aldoc | **2.951** | **2.191E-02** | | 6.644E-01 |
| 17133 | Maff | **2.941** | **1.169E-07** | | **4.070E-05** |
| 100043475 | Gm4462 | **2.901** | **2.575E-02** | | 7.082E-01 |
| 320609 | Strip2 | **2.884** | **1.159E-07** | | **4.070E-05** |
| 66808 | 9030624G23Rik | **2.877** | **3.740E-02** | | 8.326E-01 |
| 13008 | Csrp2 | **2.868** | **8.384E-04** | | 8.086E-02 |
| 23805 | Apc2 | **2.866** | **9.368E-03** | | 4.032E-01 |
| 110183 | Rn18s-rs5 | **2.864** | **1.084E-02** | | 4.404E-01 |
| 11768 | Ap1m2 | **2.849** | **3.164E-02** | | 7.741E-01 |
| 17240 | Mdfi | **2.831** | **3.493E-03** | | 2.228E-01 |
| 72575 | C430049B03Rik | **2.826** | **9.715E-03** | | 4.109E-01 |
| 102631998 | Gm30181 | **2.812** | **9.366E-03** | | 4.032E-01 |
| 100042856 | Gm4070 | **2.808** | **3.697E-02** | | 8.275E-01 |
| 108167997 | Gm46347 | **2.791** | **4.400E-03** | | 2.507E-01 |
| 108167802 | Gm46218 | **2.791** | **1.493E-02** | | 5.422E-01 |
| 234577 | Cpne2 | **2.790** | **4.654E-03** | | 2.624E-01 |
| 115490468 | LOC115490468 | **2.784** | **4.464E-02** | | 8.989E-01 |
| 14299 | Ncs1 | **2.771** | **2.817E-02** | | 7.412E-01 |
| 19699 | Reln | **2.767** | **2.329E-02** | | 6.820E-01 |
| 74272 | 1700054O19Rik | **2.761** | **1.975E-02** | | 6.319E-01 |
| 19261 | Sirpa | **2.724** | **1.838E-02** | | 6.117E-01 |
| 102632297 | Gm30409 | **2.713** | **1.871E-02** | | 6.158E-01 |
| 102639290 | Gm11963 | **2.694** | **6.904E-03** | | 3.420E-01 |
| 110454 | Ly6a | **2.682** | **7.745E-03** | | 3.647E-01 |
| 102465887 | Mir8095 | **2.681** | **4.051E-02** | | 8.590E-01 |
| 68428 | Steap3 | **2.679** | **4.138E-02** | | 8.648E-01 |
| 14345 | Fut4 | **2.672** | **1.949E-02** | | 6.267E-01 |
| 22598 | Slc6a18 | **2.669** | **2.678E-02** | | 7.257E-01 |
| 384783 | Irs2 | **2.667** | **1.177E-10** | | **9.916E-08** |
| 100042846 | Gm12057 | **2.657** | **3.630E-02** | | 8.245E-01 |
| 115487425 | LOC115487425 | **2.656** | **2.954E-02** | | 7.506E-01 |
| 108153 | Adamts7 | **2.654** | **3.252E-02** | | 7.818E-01 |
| 115487746 | LOC115487746 | **2.648** | **6.462E-03** | | 3.253E-01 |
| 100381209 | Gm17823 | **2.639** | **9.135E-03** | | 4.017E-01 |
| 100041290 | D4Ertd617e | **2.636** | **4.899E-02** | | 9.416E-01 |
| 102637465 | Gm34268 | **2.633** | **2.650E-03** | | 1.829E-01 |
| 12505 | Cd44 | **2.630** | **1.854E-04** | | **2.473E-02** |
| 18548 | Pcsk1 | **2.617** | **3.400E-06** | | **7.666E-04** |
| 230126 | Shb | **2.596** | **2.241E-02** | | 6.693E-01 |
| 12043 | Bcl2 | **2.593** | **1.951E-08** | | **8.925E-06** |
| 105244629 | Gm40210 | **2.590** | **3.766E-04** | | **4.401E-02** |
| 115489728 | LOC115489728 | **2.584** | **4.023E-03** | | 2.421E-01 |
| 330222 | Sdk1 | **2.583** | **2.425E-04** | | **3.106E-02** |
| 231834 | Snx8 | **2.538** | **3.111E-02** | | 7.650E-01 |
| 108168075 | Gm46408 | **2.526** | **4.086E-04** | | **4.640E-02** |
| 14960 | H2-Aa | **2.516** | **1.155E-02** | | 4.598E-01 |
| 115488600 | LOC115488600 | **2.513** | **4.000E-02** | | 8.590E-01 |
| 99296 | Hrh3 | **2.511** | **9.997E-03** | | 4.147E-01 |
| 108075 | Ltbp4 | **2.510** | **6.697E-03** | | 3.350E-01 |
| 432881 | Gm5466 | **2.499** | **9.308E-03** | | 4.032E-01 |
| 115490428 | LOC115490428 | **2.490** | **4.049E-02** | | 8.590E-01 |
| 78134 | Lpar4 | **2.484** | **9.317E-04** | | 8.825E-02 |
| 102634049 | Gm31728 | **2.481** | **2.293E-02** | | 6.795E-01 |
| 330409 | Cecr2 | **2.473** | **8.259E-04** | | 8.062E-02 |
| 16364 | Irf4 | **2.463** | **8.652E-05** | | **1.237E-02** |
| 15165 | Hcn1 | **2.459** | **9.925E-03** | | 4.138E-01 |
| 115486464 | LOC115486464 | **2.449** | **9.213E-03** | | 4.019E-01 |
| 100125929 | 0610009E02Rik | **2.449** | **3.537E-02** | | 8.148E-01 |
| 432530 | Adcy1 | **2.444** | **7.415E-07** | | **2.240E-04** |
| 69358 | Lrrc51 | **2.434** | **3.580E-02** | | 8.187E-01 |
| 105244435 | Gm40043 | **2.431** | **9.897E-03** | | 4.137E-01 |
| 231296 | Lrrc66 | **2.431** | **4.230E-02** | | 8.747E-01 |
| 83554 | Fstl3 | **2.429** | **1.559E-02** | | 5.608E-01 |
| 69282 | 1700001J03Rik | **2.421** | **2.776E-02** | | 7.394E-01 |
| 104099 | Itga9 | **2.420** | **9.728E-03** | | 4.109E-01 |
| 102640631 | Gm36649 | **2.417** | **3.595E-02** | | 8.199E-01 |
| 100121 | Tdrd7 | **2.395** | **3.072E-02** | | 7.621E-01 |
| 227377 | Farp2 | **2.384** | **3.685E-02** | | 8.273E-01 |
| 239319 | Card6 | **2.383** | **4.615E-02** | | 9.111E-01 |
| 17096 | Lyn | **2.382** | **1.274E-02** | | 4.940E-01 |
| 83674 | Cnnm1 | **2.380** | **4.622E-02** | | 9.112E-01 |
| 382421 | Gm5176 | **2.379** | **1.699E-02** | | 5.854E-01 |
| 100526560 | Mir3109 | **2.378** | **4.518E-02** | | 9.065E-01 |
| 209012 | Ulk4 | **2.367** | **2.524E-02** | | 7.027E-01 |
| 108169013 | Gm46894 | **2.362** | **2.179E-03** | | 1.622E-01 |
| 105352 | Dusp22 | **2.341** | **6.542E-03** | | 3.283E-01 |
| 433586 | Maml3 | **2.337** | **2.855E-05** | | **4.863E-03** |
| 105244436 | Gm37548 | **2.337** | **2.772E-08** | | **1.168E-05** |
| 115489936 | LOC115489936 | **2.330** | **5.224E-03** | | 2.842E-01 |
| 80890 | Trim2 | **2.323** | **2.366E-02** | | 6.821E-01 |
| 108168987 | Gm13205 | **2.323** | **1.949E-02** | | 6.267E-01 |
| 231510 | Gpat3 | **2.320** | **3.217E-05** | | **5.254E-03** |
| 101113 | Snx21 | **2.320** | **3.885E-02** | | 8.486E-01 |
| 22329 | Vcam1 | **2.315** | **2.601E-02** | | 7.134E-01 |
| 16768 | Lag3 | **2.301** | **4.028E-04** | | **4.639E-02** |
| 268527 | Greb1 | **2.283** | **2.083E-02** | | 6.495E-01 |
| 67260 | Cers4 | **2.283** | **7.603E-03** | | 3.612E-01 |
| 100043335 | Gm4371 | **2.280** | **3.544E-02** | | 8.152E-01 |
| 21416 | Tcf7l2 | **2.279** | **2.380E-02** | | 6.821E-01 |
| 12723 | Clcn1 | **2.264** | **1.242E-02** | | 4.861E-01 |
| 73442 | Hspa12a | **2.260** | **4.441E-02** | | 8.971E-01 |
| 70375 | Ica1l | **2.259** | **1.010E-03** | | 9.310E-02 |
| 15360 | Hmgcs2 | **2.253** | **2.224E-05** | | **3.870E-03** |
| 20430 | Cyfip1 | **2.253** | **1.481E-03** | | 1.210E-01 |
| 100417687 | Gm12846 | **2.252** | **2.439E-03** | | 1.767E-01 |
| 319939 | Tns3 | **2.246** | **1.822E-05** | | **3.353E-03** |
| 320225 | Catsperg1 | **2.244** | **3.004E-02** | | 7.564E-01 |
| 66725 | Lrrk2 | **2.235** | **3.526E-02** | | 8.145E-01 |
| 546840 | Ldlrad1 | **2.233** | **1.457E-02** | | 5.363E-01 |
| 72007 | Fndc3b | **2.233** | **6.421E-03** | | 3.253E-01 |
| 16149 | Cd74 | **2.232** | **4.101E-03** | | 2.450E-01 |
| 17476 | Mpeg1 | **2.230** | **4.397E-02** | | 8.955E-01 |
| 102637099 | Gm38495 | **2.224** | **1.562E-03** | | 1.256E-01 |
| 115486901 | LOC115486901 | **2.220** | **1.479E-03** | | 1.210E-01 |
| 108168376 | Gm46620 | **2.214** | **6.210E-03** | | 3.176E-01 |
| 108168205 | Gm46489 | **2.209** | **3.251E-02** | | 7.818E-01 |
| 13992 | Khdrbs3 | **2.198** | **2.875E-02** | | 7.443E-01 |
| 101809 | Spred3 | **2.197** | **1.814E-02** | | 6.063E-01 |
| 319743 | 9630013D21Rik | **2.192** | **8.822E-03** | | 3.923E-01 |
| 21873 | Tjp2 | **2.190** | **1.419E-02** | | 5.319E-01 |
| 12260 | C1qb | **2.183** | **3.747E-02** | | 8.326E-01 |
| 100503470 | Gm16619 | **2.180** | **1.688E-02** | | 5.843E-01 |
| 229715 | Amigo1 | **2.173** | **3.729E-03** | | 2.320E-01 |
| 17181 | Matn2 | **2.170** | **1.478E-03** | | 1.210E-01 |
| 107815 | Scml2 | **2.159** | **1.452E-02** | | 5.361E-01 |
| 102634592 | Gm32133 | **2.153** | **4.216E-02** | | 8.744E-01 |
| 102640410 | Gm36475 | **2.148** | **2.802E-02** | | 7.412E-01 |
| 208727 | Hdac4 | **2.147** | **4.049E-06** | | **8.881E-04** |
| 77411 | Esrp2 | **2.144** | **3.784E-05** | | **6.119E-03** |
| 73094 | Sgip1 | **2.140** | **4.393E-03** | | 2.507E-01 |
| 108167732 | Gm46162 | **2.139** | **2.105E-02** | | 6.532E-01 |
| 13982 | Esr1 | **2.131** | **4.263E-03** | | 2.502E-01 |
| 100764 | Rita1 | **2.129** | **1.179E-03** | | 1.037E-01 |
| 71302 | Arhgap26 | **2.092** | **2.090E-05** | | **3.677E-03** |
| 386454 | Rnf39 | **2.073** | **3.425E-02** | | 8.054E-01 |
| 433520 | Gm14403 | **2.064** | **1.884E-02** | | 6.158E-01 |
| 110197 | Dgkg | **2.056** | **6.982E-07** | | **2.149E-04** |
| 67425 | Eps8l1 | **2.055** | **1.056E-03** | | 9.553E-02 |
| 52055 | Rab11fip5 | **2.050** | **3.300E-02** | | 7.896E-01 |
| 208080 | Ubap1l | **2.046** | **1.680E-02** | | 5.843E-01 |
| 621304 | Gm6209 | **2.044** | **7.908E-04** | | 7.863E-02 |
| 18563 | Pcx | **2.036** | **1.363E-02** | | 5.185E-01 |
| 226751 | Cdc42bpa | **2.036** | **4.267E-03** | | 2.502E-01 |
| 18029 | Nfic | **2.035** | **1.409E-02** | | 5.310E-01 |
| 71354 | Wdr31 | **2.029** | **2.828E-02** | | 7.421E-01 |
| 108167659 | LOC108167659 | **2.025** | **7.957E-03** | | 3.683E-01 |
| 667481 | Trav6d-5 | **2.023** | **9.011E-03** | | 3.996E-01 |
| 19250 | Ptpn14 | **2.012** | **3.151E-06** | | **7.312E-04** |
| 360213 | Trim46 | **2.002** | **1.690E-02** | | 5.843E-01 |
| 229731 | Slc25a24 | **2.001** | **1.313E-03** | | 1.100E-01 |
| 399591 | Tmsb15l | **-2.018** | **4.308E-03** | | 2.507E-01 |
| 20620 | Plk2 | **-2.025** | **4.574E-02** | | 9.111E-01 |
| 74050 | 4921525O09Rik | **-2.028** | **4.669E-04** | | 5.155E-02 |
| 56043 | Akr1e1 | **-2.030** | **3.107E-02** | | 7.650E-01 |
| 17064 | Cd93 | **-2.031** | **2.832E-02** | | 7.421E-01 |
| 102637967 | Gm34647 | **-2.040** | **4.912E-02** | | 9.417E-01 |
| 23969 | Pacsin1 | **-2.042** | **9.998E-03** | | 4.147E-01 |
| 115490493 | LOC115490493 | **-2.056** | **3.644E-02** | | 8.245E-01 |
| 50785 | Hs6st1 | **-2.061** | **1.439E-06** | | **3.808E-04** |
| 65945 | Clstn1 | **-2.063** | **2.820E-02** | | 7.412E-01 |
| 240120 | Zfp119b | **-2.081** | **3.489E-02** | | 8.090E-01 |
| 17724 | mt-Rnr1 | **-2.088** | **1.999E-02** | | 6.336E-01 |
| 67775 | Rtp4 | **-2.091** | **2.213E-02** | | 6.685E-01 |
| 68992 | Zfp580 | **-2.092** | **4.083E-02** | | 8.623E-01 |
| 71091 | Cdkl1 | **-2.094** | **5.211E-03** | | 2.842E-01 |
| 15024 | H2-T10 | **-2.106** | **3.059E-05** | | **5.102E-03** |
| 381417 | Gm14085 | **-2.112** | **1.013E-05** | | **2.027E-03** |
| 115487084 | LOC115487084 | **-2.119** | **3.085E-02** | | 7.623E-01 |
| 19331 | Rab19 | **-2.122** | **5.392E-04** | | 5.754E-02 |
| 75782 | Lca5 | **-2.130** | **1.602E-04** | | **2.155E-02** |
| 14168 | Fgf13 | **-2.131** | **1.959E-03** | | 1.508E-01 |
| 59010 | Sqor | **-2.134** | **5.816E-05** | | **8.867E-03** |
| 115488064 | LOC115488064 | **-2.135** | **3.551E-02** | | 8.156E-01 |
| 52076 | Tmem38b | **-2.138** | **1.760E-02** | | 5.958E-01 |
| 233552 | Gdpd5 | **-2.144** | **3.379E-02** | | 7.993E-01 |
| 73991 | Atl1 | **-2.155** | **2.697E-02** | | 7.271E-01 |
| 115490050 | LOC115490050 | **-2.156** | **3.258E-02** | | 7.820E-01 |
| 58220 | Pard6b | **-2.163** | **1.323E-02** | | 5.066E-01 |
| 81913 | Bambi-ps1 | **-2.164** | **2.046E-05** | | **3.639E-03** |
| 107022 | Gramd3 | **-2.165** | **2.744E-04** | | **3.405E-02** |
| 102640217 | Gm36338 | **-2.167** | **4.234E-02** | | 8.747E-01 |
| 666348 | Apol7e | **-2.168** | **4.025E-04** | | **4.639E-02** |
| 105242677 | Gm38828 | **-2.170** | **3.448E-03** | | 2.208E-01 |
| 72121 | Dennd2d | **-2.183** | **9.243E-06** | | **1.873E-03** |
| 14114 | Fbln1 | **-2.183** | **8.037E-03** | | 3.692E-01 |
| 100217422 | Snord13 | **-2.195** | **2.435E-02** | | 6.899E-01 |
| 100034675 | Gm11335 | **-2.205** | **3.198E-02** | | 7.769E-01 |
| 102632039 | Gm30214 | **-2.219** | **4.017E-02** | | 8.590E-01 |
| 654824 | Ankrd37 | **-2.221** | **3.175E-03** | | 2.066E-01 |
| 664779 | Gm44505 | **-2.229** | **9.247E-03** | | 4.023E-01 |
| 217882 | Cep170b | **-2.244** | **7.412E-03** | | 3.553E-01 |
| 77032 | Tstd3 | **-2.248** | **2.370E-02** | | 6.821E-01 |
| 386655 | Eid2 | **-2.250** | **3.800E-02** | | 8.360E-01 |
| 414094 | Dgkeos | **-2.255** | **1.861E-05** | | **3.386E-03** |
| 108637 | Snord14c | **-2.255** | **2.028E-02** | | 6.390E-01 |
| 140577 | Ankrd6 | **-2.261** | **9.148E-06** | | **1.873E-03** |
| 242126 | Slc22a15 | **-2.264** | **4.738E-03** | | 2.643E-01 |
| 278679 | Apol7b | **-2.269** | **3.760E-03** | | 2.324E-01 |
| 56325 | Abcb9 | **-2.271** | **2.977E-06** | | **7.009E-04** |
| 21942 | Tnfrsf9 | **-2.294** | **3.844E-02** | | 8.440E-01 |
| 20408 | Sh3gl3 | **-2.302** | **1.387E-02** | | 5.262E-01 |
| 115486004 | LOC115486004 | **-2.302** | **3.803E-03** | | 2.333E-01 |
| 102633785 | Gm31527 | **-2.305** | **2.330E-02** | | 6.820E-01 |
| 106952 | Arap3 | **-2.305** | **3.418E-02** | | 8.054E-01 |
| 22436 | Xdh | **-2.308** | **3.326E-03** | | 2.156E-01 |
| 108167368 | LOC108167368 | **-2.312** | **3.043E-02** | | 7.613E-01 |
| 19122 | Prnp | **-2.312** | **5.151E-04** | | 5.610E-02 |
| 102637188 | Gm34065 | **-2.323** | **2.376E-02** | | 6.821E-01 |
| 14073 | Faah | **-2.324** | **1.904E-05** | | **3.424E-03** |
| 115487687 | LOC115487687 | **-2.328** | **1.136E-05** | | **2.218E-03** |
| 241489 | Pde11a | **-2.334** | **6.455E-03** | | 3.253E-01 |
| 115487522 | LOC115487522 | **-2.335** | **5.152E-03** | | 2.824E-01 |
| 214230 | Pak6 | **-2.340** | **2.533E-03** | | 1.774E-01 |
| 67252 | Cap2 | **-2.344** | **1.734E-02** | | 5.912E-01 |
| 105246174 | Gm41508 | **-2.349** | **2.885E-02** | | 7.450E-01 |
| 16574 | Kif5c | **-2.356** | **2.927E-02** | | 7.504E-01 |
| 211027 | Trav3-4 | **-2.360** | **1.540E-02** | | 5.554E-01 |
| 666244 | Tmsb15b1 | **-2.369** | **1.895E-02** | | 6.178E-01 |
| 76220 | 6530402F18Rik | **-2.369** | **1.070E-02** | | 4.368E-01 |
| 666240 | Gm8000 | **-2.369** | **3.559E-02** | | 8.162E-01 |
| 665367 | Gm12350 | **-2.372** | **3.789E-02** | | 8.360E-01 |
| 115486514 | LOC115486514 | **-2.410** | **3.692E-02** | | 8.275E-01 |
| 100039355 | Rps16-ps2 | **-2.414** | **4.396E-03** | | 2.507E-01 |
| 57440 | Ehd3 | **-2.418** | **2.246E-06** | | **5.619E-04** |
| 378937 | Lrrc24 | **-2.421** | **3.877E-02** | | 8.480E-01 |
| 53314 | Batf | **-2.426** | **1.827E-03** | | 1.441E-01 |
| 12819 | Col15a1 | **-2.452** | **7.498E-03** | | 3.583E-01 |
| 83671 | Sytl2 | **-2.452** | **7.192E-04** | | 7.287E-02 |
| 74466 | Mfsd13b | **-2.453** | **3.026E-02** | | 7.592E-01 |
| 140488 | Igf2bp3 | **-2.481** | **9.048E-04** | | 8.622E-02 |
| 51789 | Tnk2 | **-2.487** | **3.358E-09** | | **1.720E-06** |
| 102633360 | Gm31204 | **-2.489** | **2.243E-02** | | 6.693E-01 |
| 102633439 | Gm14154 | **-2.501** | **2.524E-03** | | 1.774E-01 |
| 100042235 | Gm3739 | **-2.501** | **4.285E-03** | | 2.504E-01 |
| 15504 | Dnajb3 | **-2.522** | **4.160E-02** | | 8.675E-01 |
| 73379 | Dcbld2 | **-2.531** | **2.724E-02** | | 7.288E-01 |
| 595140 | Gm6044 | **-2.547** | **4.954E-02** | | 9.434E-01 |
| 65116 | Prrg2 | **-2.551** | **2.723E-02** | | 7.288E-01 |
| 102636372 | Gm33457 | **-2.560** | **2.871E-02** | | 7.443E-01 |
| 115488085 | LOC115488085 | **-2.562** | **3.005E-02** | | 7.564E-01 |
| 240066 | Zfp870 | **-2.574** | **1.487E-02** | | 5.418E-01 |
| 229672 | Bcl2l15 | **-2.581** | **1.912E-02** | | 6.222E-01 |
| 20660 | Sorl1 | **-2.584** | **1.569E-04** | | **2.129E-02** |
| 102636200 | Gm33335 | **-2.591** | **1.969E-02** | | 6.316E-01 |
| 243374 | Gimap8 | **-2.598** | **2.949E-09** | | **1.574E-06** |
| 12608 | Cebpb | **-2.620** | **3.078E-03** | | 2.036E-01 |
| 22351 | Vill | **-2.621** | **2.170E-02** | | 6.629E-01 |
| 20667 | Sox12 | **-2.625** | **2.301E-02** | | 6.795E-01 |
| 100101942 | Trdv2-1 | **-2.629** | **3.454E-02** | | 8.060E-01 |
| 13819 | Epas1 | **-2.629** | **2.807E-02** | | 7.412E-01 |
| 102638019 | Gm34687 | **-2.634** | **2.435E-02** | | 6.899E-01 |
| 115487892 | LOC115487892 | **-2.635** | **1.223E-06** | | **3.374E-04** |
| 102640377 | Gm36449 | **-2.642** | **1.991E-02** | | 6.325E-01 |
| 67795 | Rnls | **-2.648** | **3.266E-02** | | 7.827E-01 |
| 18559 | Pctp | **-2.655** | **8.587E-03** | | 3.870E-01 |
| 70080 | Igsf23 | **-2.664** | **2.255E-04** | | **2.911E-02** |
| 115487690 | LOC115487690 | **-2.666** | **6.614E-05** | | **9.896E-03** |
| 102631948 | Gm30149 | **-2.689** | **7.959E-03** | | 3.683E-01 |
| 12772 | Ccr2 | **-2.691** | **4.015E-03** | | 2.421E-01 |
| 102635138 | Gm38474 | **-2.701** | **3.000E-02** | | 7.564E-01 |
| 77428 | 9430083A17Rik | **-2.714** | **1.978E-02** | | 6.319E-01 |
| 626578 | Gbp10 | **-2.760** | **7.743E-07** | | **2.295E-04** |
| 332579 | Card9 | **-2.796** | **1.022E-06** | | **2.870E-04** |
| 100503915 | Smpd5 | **-2.798** | **7.376E-03** | | 3.546E-01 |
| 105245263 | Gm40741 | **-2.804** | **2.308E-03** | | 1.687E-01 |
| 224129 | Adcy5 | **-2.808** | **1.297E-02** | | 5.016E-01 |
| 115490291 | LOC115490291 | **-2.813** | **3.184E-02** | | 7.758E-01 |
| 105841 | Dennd3 | **-2.832** | **1.102E-03** | | 9.852E-02 |
| 76469 | Cmya5 | **-2.839** | **8.853E-05** | | **1.254E-02** |
| 108168823 | Gm46781 | **-2.868** | **3.324E-02** | | 7.907E-01 |
| 330483 | Ceacam16 | **-2.877** | **2.047E-02** | | 6.413E-01 |
| 105243145 | Gm39145 | **-2.878** | **1.453E-02** | | 5.361E-01 |
| 115489019 | LOC115489019 | **-2.885** | **1.466E-02** | | 5.365E-01 |
| 229665 | Ampd1 | **-2.907** | **4.860E-05** | | **7.628E-03** |
| 73919 | Lyrm1 | **-2.912** | **9.796E-03** | | 4.116E-01 |
| 21753 | Tes | **-2.955** | **4.173E-08** | | **1.590E-05** |
| 26458 | Slc27a2 | **-2.961** | **1.853E-02** | | 6.140E-01 |
| 115488025 | LOC115488025 | **-2.962** | **4.017E-02** | | 8.590E-01 |
| 102640419 | Gm16322 | **-2.967** | **1.539E-02** | | 5.554E-01 |
| 75744 | Svip | **-3.005** | **1.944E-02** | | 6.267E-01 |
| 115490087 | LOC115490087 | **-3.030** | **3.103E-02** | | 7.650E-01 |
| 230379 | Acer2 | **-3.039** | **1.052E-02** | | 4.308E-01 |
| 11981 | Atp9a | **-3.049** | **6.786E-03** | | 3.384E-01 |
| 56643 | Slc15a1 | **-3.057** | **6.942E-03** | | 3.420E-01 |
| 15208 | Hes5 | **-3.061** | **2.218E-02** | | 6.686E-01 |
| 100702 | Gbp6 | **-3.070** | **2.303E-12** | | **2.634E-09** |
| 100041622 | Gm3436 | **-3.070** | **3.597E-02** | | 8.199E-01 |
| 218763 | Lrrc3b | **-3.076** | **8.003E-03** | | 3.692E-01 |
| 64113 | Moap1 | **-3.081** | **2.332E-02** | | 6.820E-01 |
| 666031 | Gm7899 | **-3.115** | **1.770E-02** | | 5.971E-01 |
| 11490 | Adam15 | **-3.121** | **7.814E-03** | | 3.655E-01 |
| 100041925 | Gm14586 | **-3.141** | **1.743E-02** | | 5.913E-01 |
| 105246085 | Gm41432 | **-3.170** | **5.933E-03** | | 3.084E-01 |
| 70727 | Rasgef1a | **-3.174** | **1.410E-02** | | 5.310E-01 |
| 19023 | Ppef2 | **-3.199** | **1.264E-02** | | 4.924E-01 |
| 225825 | Cd226 | **-3.212** | **1.666E-05** | | **3.213E-03** |
| 13052 | Cxadr | **-3.224** | **1.698E-03** | | 1.352E-01 |
| 442803 | A830005F24Rik | **-3.241** | **9.595E-03** | | 4.085E-01 |
| 16400 | Itga3 | **-3.265** | **3.683E-03** | | 2.320E-01 |
| 20440 | St6gal1 | **-3.267** | **1.965E-09** | | **1.123E-06** |
| 20613 | Snai1 | **-3.271** | **8.051E-03** | | 3.692E-01 |
| 105242589 | Gm38759 | **-3.287** | **9.572E-03** | | 4.085E-01 |
| 225392 | Rell2 | **-3.289** | **7.206E-03** | | 3.490E-01 |
| 115489965 | LOC115489965 | **-3.300** | **3.934E-03** | | 2.403E-01 |
| 115489981 | LOC115489981 | **-3.346** | **7.441E-04** | | 7.445E-02 |
| 14200 | Fhl2 | **-3.452** | **5.904E-04** | | 6.218E-02 |
| 71141 | 4933407L21Rik | **-3.523** | **2.947E-02** | | 7.506E-01 |
| 12877 | Cpeb1 | **-3.543** | **2.726E-05** | | **4.692E-03** |
| 280047 | Gm5075 | **-3.546** | **3.977E-02** | | 8.590E-01 |
| 58233 | Dnaja4 | **-3.550** | **2.198E-02** | | 6.651E-01 |
| 243923 | Rgs9bp | **-3.590** | **1.875E-02** | | 6.158E-01 |
| 70839 | P2ry12 | **-3.591** | **1.272E-02** | | 4.940E-01 |
| 434782 | Gm5637 | **-3.609** | **4.771E-02** | | 9.247E-01 |
| 70821 | 4921507P07Rik | **-3.632** | **2.381E-02** | | 6.821E-01 |
| 100043225 | Gm16378 | **-3.669** | **2.796E-02** | | 7.412E-01 |
| 102631731 | Gm29998 | **-3.696** | **2.670E-03** | | 1.835E-01 |
| 71981 | Tdrd12 | **-3.703** | **3.707E-02** | | 8.277E-01 |
| 433739 | Gm12799 | **-3.743** | **1.335E-02** | | 5.102E-01 |
| 77794 | Adamtsl2 | **-3.753** | **1.420E-07** | | **4.737E-05** |
| 22055 | n-THgtg3 | **-3.767** | **4.868E-02** | | 9.390E-01 |
| 102634915 | Gm12268 | **-3.789** | **1.452E-02** | | 5.361E-01 |
| 620772 | Gm6180 | **-3.793** | **2.179E-02** | | 6.631E-01 |
| 100043843 | Rps15a-ps7 | **-3.829** | **3.055E-02** | | 7.617E-01 |
| 381404 | Pabpc1l | **-3.866** | **2.215E-03** | | 1.642E-01 |
| 115488237 | LOC115488237 | **-3.867** | **1.989E-03** | | 1.523E-01 |
| 12671 | Chrm3 | **-3.906** | **1.808E-02** | | 6.054E-01 |
| 105245236 | LOC105245236 | **-3.947** | **3.321E-02** | | 7.907E-01 |
| 18762 | Prkcz | **-4.008** | **3.257E-08** | | **1.304E-05** |
| 433938 | Mn1 | **-4.012** | **3.134E-03** | | 2.051E-01 |
| 100862247 | Gm21586 | **-4.036** | **1.301E-02** | | 5.019E-01 |
| 16776 | Lama5 | **-4.056** | **1.382E-03** | | 1.153E-01 |
| 218695 | Gm10044 | **-4.137** | **1.178E-02** | | 4.675E-01 |
| 18227 | Nr4a2 | **-4.179** | **2.172E-03** | | 1.622E-01 |
| 115487436 | LOC115487436 | **-4.193** | **2.603E-02** | | 7.134E-01 |
| 108167895 | Gm46277 | **-4.219** | **2.140E-09** | | **1.181E-06** |
| 115487786 | LOC115487786 | **-4.287** | **4.114E-02** | | 8.624E-01 |
| 105193 | Nhlrc1 | **-4.296** | **1.209E-02** | | 4.742E-01 |
| 74901 | Kbtbd11 | **-4.308** | **3.885E-12** | | **3.887E-09** |
| 231603 | A630023P12Rik | **-4.324** | **2.491E-03** | | 1.772E-01 |
| 433070 | Vmn2r96 | **-4.328** | **1.096E-03** | | 9.852E-02 |
| 22652 | Mkrn3 | **-4.360** | **2.517E-02** | | 7.019E-01 |
| 231932 | Gimap7 | **-4.468** | **2.681E-06** | | **6.504E-04** |
| 665004 | Gm7443 | **-4.471** | **1.095E-02** | | 4.426E-01 |
| 14702 | Gng2 | **-4.583** | **3.692E-04** | | **4.346E-02** |
| 329628 | Fat4 | **-4.596** | **6.037E-05** | | **9.118E-03** |
| 108168021 | Gm46370 | **-4.616** | **2.986E-02** | | 7.564E-01 |
| 102636184 | Gm33323 | **-4.629** | **9.653E-04** | | 9.037E-02 |
| 100416808 | Zfp422-ps | **-4.810** | **2.186E-02** | | 6.639E-01 |
| 676984 | Gm9701 | **-4.813** | **5.389E-03** | | 2.886E-01 |
| 272009 | Srsf12 | **-4.853** | **4.080E-04** | | **4.640E-02** |
| 667977 | Gm8909 | **-5.021** | **2.470E-08** | | **1.098E-05** |
| 218476 | Gcnt4 | **-5.060** | **1.798E-05** | | **3.346E-03** |
| 115488400 | LOC115488400 | **-5.669** | **2.503E-02** | | 7.018E-01 |
| 319574 | Gm20735 | **-5.729** | **2.727E-03** | | 1.866E-01 |
| 20564 | Slit3 | **-5.827** | **1.230E-04** | | **1.727E-02** |
| 12672 | Chrm4 | **-5.903** | **3.434E-03** | | 2.208E-01 |
| 69717 | Gm10499 | **-6.067** | **1.221E-11** | | **1.086E-08** |
| 387134 | Mir16-1 | **-6.070** | **2.503E-02** | | 7.018E-01 |
| 102467530 | n-TGgcc2 | **-6.671** | **4.451E-03** | | 2.527E-01 |
| 15530 | Hspg2 | **-6.799** | **2.943E-12** | | **3.141E-09** |
| 50930 | Tnfsf14 | **-7.277** | **1.343E-10** | | **1.075E-07** |
| 115487043 | LOC115487043 | **-7.728** | **6.346E-03** | | 3.235E-01 |
| 16336 | Insl3 | **-7.905** | **2.019E-02** | | 6.389E-01 |
| 259277 | Klk8 | **-8.308** | **1.819E-06** | | **4.697E-04** |
| 100736249 | Mira | **-8.640** | **2.164E-04** | | **2.817E-02** |
| 102634419 | Gm32004 | **-8.899** | **1.230E-19** | | **3.281E-16** |
| 102634148 | Gm31803 | **-10.816** | **1.129E-11** | | **1.064E-08** |
| 12895 | Cpt1b | **-14.440** | **1.430E-15** | | **1.908E-12** |
| 115485636 | LOC115485636 | **-16.598** | **1.294E-03** | | 1.096E-01 |
