# Supplementary material for: Regulation of positive and negative selection and TCR signaling during thymic T cell development by capicua
Source: eLife. 2021 Dec 13;10:e71769. doi: 10.7554/eLife.71769 (PMC8700290; doi:10.7554/eLife.71769)
Supplement: Supplementary file 2. [file elife-71769-supp2.docx]

| **Table S2-1. Gene Ontology (GO) analysis of the DEGs in *Cic*-deficient DP thymocytes (upregulated DEGs)** | | | | | | | | | | | | |
| --- | --- | --- | --- | --- | --- | --- | --- | --- | --- | --- | --- | --- |
| Category | Term | Count | % | PValue | Genes | List Total | Pop Hits | Pop Total | Fold Enrichment | Bonferroni | Benjamini | FDR |
| GOTERM_BP_DIRECT | GO:0071277~cellular response to calcium ion | 6 | 2.586 | 0.000 | TRPM2, MEF2C, CPNE2, ADCY1, SCN5A, SLC25A24 | 145 | 53 | 18082 | 14.117 | 0.071 | 0.074 | 0.074 |
| GOTERM_BP_DIRECT | GO:0000188~inactivation of MAPK activity | 4 | 1.724 | 0.000 | SPRED3, DUSP4, SPRED1, DUSP6 | 145 | 15 | 18082 | 33.254 | 0.218 | 0.123 | 0.122 |
| GOTERM_BP_DIRECT | GO:0016477~cell migration | 8 | 3.448 | 0.001 | APC2, STRIP2, RELN, FNDC3B, SIRPA, CDC42BPA, TNS3, CD44 | 145 | 191 | 18082 | 5.223 | 0.633 | 0.241 | 0.240 |
| GOTERM_BP_DIRECT | GO:0051966~regulation of synaptic transmission, glutamatergic | 4 | 1.724 | 0.001 | GRIA2, MEF2C, SERPINE2, LRRK2 | 145 | 24 | 18082 | 20.784 | 0.646 | 0.241 | 0.240 |
| GOTERM_BP_DIRECT | GO:0030178~negative regulation of Wnt signaling pathway | 5 | 2.155 | 0.001 | APC2, LRP1, CCND1, MDFI, DACT1 | 145 | 56 | 18082 | 11.134 | 0.700 | 0.241 | 0.240 |
| GOTERM_BP_DIRECT | GO:0007275~multicellular organism development | 19 | 8.190 | 0.001 | DLX1, CYFIP1, MEF2C, PKDCC, LRP1, SERPINE2, FZD7, AMIGO1, SPRY4, TSHZ2, TDRD7, SHB, SPRED3, SPRED1, CSRP2, RELN, NKX2-4, MDFI, DACT1 | 145 | 1029 | 18082 | 2.303 | 0.803 | 0.270 | 0.269 |
| GOTERM_BP_DIRECT | GO:0008283~cell proliferation | 8 | 3.448 | 0.002 | APC2, CSF1R, CD74, TCF7L2, LRP1, BCL2, IRS2, APPL2 | 145 | 220 | 18082 | 4.535 | 0.896 | 0.323 | 0.322 |
| GOTERM_BP_DIRECT | GO:0043523~regulation of neuron apoptotic process | 4 | 1.724 | 0.002 | MEF2C, TRIM2, KCNIP3, ESR1 | 145 | 33 | 18082 | 15.116 | 0.930 | 0.332 | 0.330 |
| GOTERM_BP_DIRECT | GO:0050679~positive regulation of epithelial cell proliferation | 5 | 2.155 | 0.003 | TCF7L2, HRH3, ESRP2, SCN5A, ESR1 | 145 | 76 | 18082 | 8.204 | 0.976 | 0.413 | 0.412 |
| GOTERM_BP_DIRECT | GO:0034765~regulation of ion transmembrane transport | 6 | 2.586 | 0.005 | KCNIP3, KCNJ16, SCN5A, CLCN1, CACNG4, HCN1 | 145 | 135 | 18082 | 5.542 | 0.995 | 0.472 | 0.470 |
| GOTERM_BP_DIRECT | GO:0070932~histone H3 deacetylation | 3 | 1.293 | 0.005 | PER2, HDAC4, HDAC9 | 145 | 13 | 18082 | 28.778 | 0.996 | 0.472 | 0.470 |
| GOTERM_BP_DIRECT | GO:0030890~positive regulation of B cell proliferation | 4 | 1.724 | 0.005 | CD74, MEF2C, BCL2, IRS2 | 145 | 43 | 18082 | 11.600 | 0.997 | 0.472 | 0.470 |
| GOTERM_BP_DIRECT | GO:0006811~ion transport | 12 | 5.172 | 0.007 | TRPM2, GRIA2, STEAP3, SLC5A9, CNNM1, STEAP4, KCNIP3, KCNJ16, SCN5A, CLCN1, CACNG4, HCN1 | 145 | 584 | 18082 | 2.562 | 1.000 | 0.607 | 0.605 |
| GOTERM_BP_DIRECT | GO:0019882~antigen processing and presentation | 4 | 1.724 | 0.009 | CD74, RAB4A, H2-AA, CTSS | 145 | 54 | 18082 | 9.237 | 1.000 | 0.607 | 0.605 |
| GOTERM_BP_DIRECT | GO:0030154~cell differentiation | 14 | 6.034 | 0.010 | DLX1, CYFIP1, MEF2C, PKDCC, SERPINE2, LRRK2, AMIGO1, CHD5, TDRD7, SHB, DUSP6, ETV5, CSRP2, MDFI | 145 | 780 | 18082 | 2.238 | 1.000 | 0.607 | 0.605 |

| **Table S2-2. Gene Ontology (GO) analysis of the DEGs in Cic-deficient DP thymocytes (downregulated DEGs)** | | | | | | | | | | | | |
| --- | --- | --- | --- | --- | --- | --- | --- | --- | --- | --- | --- | --- |
| Category | Term | Count | % | PValue | Genes | List Total | Pop Hits | Pop Total | Fold Enrichment | Bonferroni | Benjamini | FDR |
| GOTERM_BP_DIRECT | GO:0007613~memory | 6 | 3.141 | 0.000 | CEBPB, ITGA3, PLK2, PAK6, FGF13, KLK8 | 113 | 83 | 18082 | 11.568 | 0.114 | 0.121 | 0.121 |
| GOTERM_BP_DIRECT | GO:0007155~cell adhesion | 10 | 5.236 | 0.003 | LAMA5, COL15A1, CXADR, ADAM15, CD93, ITGA3, CLSTN1, CD226, FAT4, HES5 | 113 | 485 | 18082 | 3.299 | 0.905 | 1.000 | 1.000 |
| GOTERM_BP_DIRECT | GO:0016477~cell migration | 6 | 3.141 | 0.007 | LAMA5, RASGEF1A, TNK2, SNAI1, SORL1, PRKCZ | 113 | 191 | 18082 | 5.027 | 0.993 | 1.000 | 1.000 |
| GOTERM_BP_DIRECT | GO:0042832~defense response to protozoan | 3 | 1.571 | 0.015 | GBP6, GBP10, BATF | 113 | 30 | 18082 | 16.002 | 1.000 | 1.000 | 1.000 |
| GOTERM_BP_DIRECT | GO:0045408~regulation of interleukin-6 biosynthetic process | 2 | 1.047 | 0.018 | CEBPB, CARD9 | 113 | 3 | 18082 | 106.678 | 1.000 | 1.000 | 1.000 |
| GOTERM_BP_DIRECT | GO:0006897~endocytosis | 5 | 2.618 | 0.026 | SH3GL3, TNK2, SORL1, ATP9A, PACSIN1 | 113 | 181 | 18082 | 4.420 | 1.000 | 1.000 | 1.000 |
| GOTERM_BP_DIRECT | GO:0070885~negative regulation of calcineurin-NFAT signaling cascade | 2 | 1.047 | 0.031 | PRNP, CMYA5 | 113 | 5 | 18082 | 64.007 | 1.000 | 1.000 | 1.000 |
| GOTERM_BP_DIRECT | GO:0006869~lipid transport | 4 | 2.094 | 0.032 | APOL7B, PCTP, SORL1, APOL7E | 113 | 111 | 18082 | 5.766 | 1.000 | 1.000 | 1.000 |
| GOTERM_BP_DIRECT | GO:0030198~extracellular matrix organization | 4 | 2.094 | 0.034 | LAMA5, ADAMTSL2, FBLN1, HSPG2 | 113 | 114 | 18082 | 5.615 | 1.000 | 1.000 | 1.000 |
| GOTERM_BP_DIRECT | GO:0007157~heterophilic cell-cell adhesion via plasma membrane cell adhesion molecules | 3 | 1.571 | 0.041 | CXADR, CD226, FAT4 | 113 | 52 | 18082 | 9.232 | 1.000 | 1.000 | 1.000 |
| GOTERM_BP_DIRECT | GO:0007197~adenylate cyclase-inhibiting G-protein coupled acetylcholine receptor signaling pathway | 2 | 1.047 | 0.043 | CHRM3, CHRM4 | 113 | 7 | 18082 | 45.719 | 1.000 | 1.000 | 1.000 |
| GOTERM_BP_DIRECT | GO:0007207~phospholipase C-activating G-protein coupled acetylcholine receptor signaling pathway | 2 | 1.047 | 0.043 | CHRM3, CHRM4 | 113 | 7 | 18082 | 45.719 | 1.000 | 1.000 | 1.000 |
| GOTERM_BP_DIRECT | GO:0001973~adenosine receptor signaling pathway | 2 | 1.047 | 0.049 | P2RY12, ADCY5 | 113 | 8 | 18082 | 40.004 | 1.000 | 1.000 | 1.000 |
| GOTERM_BP_DIRECT | GO:0072006~nephron development | 2 | 1.047 | 0.049 | ITGA3, FAT4 | 113 | 8 | 18082 | 40.004 | 1.000 | 1.000 | 1.000 |
| GOTERM_BP_DIRECT | GO:0015833~peptide transport | 2 | 1.047 | 0.049 | SLC15A1, ABCB9 | 113 | 8 | 18082 | 40.004 | 1.000 | 1.000 | 1.000 |
