# Supplementary material for: Regulation of positive and negative selection and TCR signaling during thymic T cell development by capicua
Source: eLife. 2021 Dec 13;10:e71769. doi: 10.7554/eLife.71769 (PMC8700290; doi:10.7554/eLife.71769)
Supplement: Supplementary file 3. [file elife-71769-supp3.docx]

| **Table S3. Oligonucleotide sequences used for qRT-PCR** | |
| --- | --- |
| Primer name | Sequences |
| Bcl2 (Forward) | 5ʹ-ATGCCTTTGTGGAACTATATGGC-3ʹ |
| Bcl2 (Reverse) | 5ʹ-GGTATGCACCCAGAGTGATGC-3 |
| Spry4 (Forward) | 5ʹ-GCAGCGTCCCTGTGAATCC-3ʹ |
| Spry4 (Reverse) | 5ʹ-TCTGGTCAATGGGTAAGATGGT-3ʹ |
| Dusp4 (Forward) | 5ʹ-TCCCCGTCGAAGACAACCA-3ʹ |
| Dusp4 (Reverse) | 5ʹ-CTTTACTGCGTCGATGTACTCG-3ʹ |
| Dusp6 (Forward) | 5ʹ-ATAGATACGCTCAGACCCGTG-3ʹ |
| Dusp6 (Reverse) | 5ʹ-ATCAGCAGAAGCCGTTCGTT-3ʹ |
| Spred1 (Forward) | 5ʹ-GAGATGACTCAAGTGGTGGATG-3’ |
| Spred1 (Reverse) | 5ʹ- TCTGAAAGGTAAGGCCAAACTTC-3ʹ |
| Hprt (Forward) | 5ʹ-TCAGTCAACGGGGGACATAAA-3ʹ |
| Hprt (Reverse) | 5ʹ- GGGGCTGTACTGCTTAACCAG-3ʹ |
