# Supplementary material for: Regulation of positive and negative selection and TCR signaling during thymic T cell development by capicua
Source: eLife. 2021 Dec 13;10:e71769. doi: 10.7554/eLife.71769 (PMC8700290; doi:10.7554/eLife.71769)
Supplement: Supplementary file 4. [file elife-71769-supp4.pdf]

Supplementary file 4

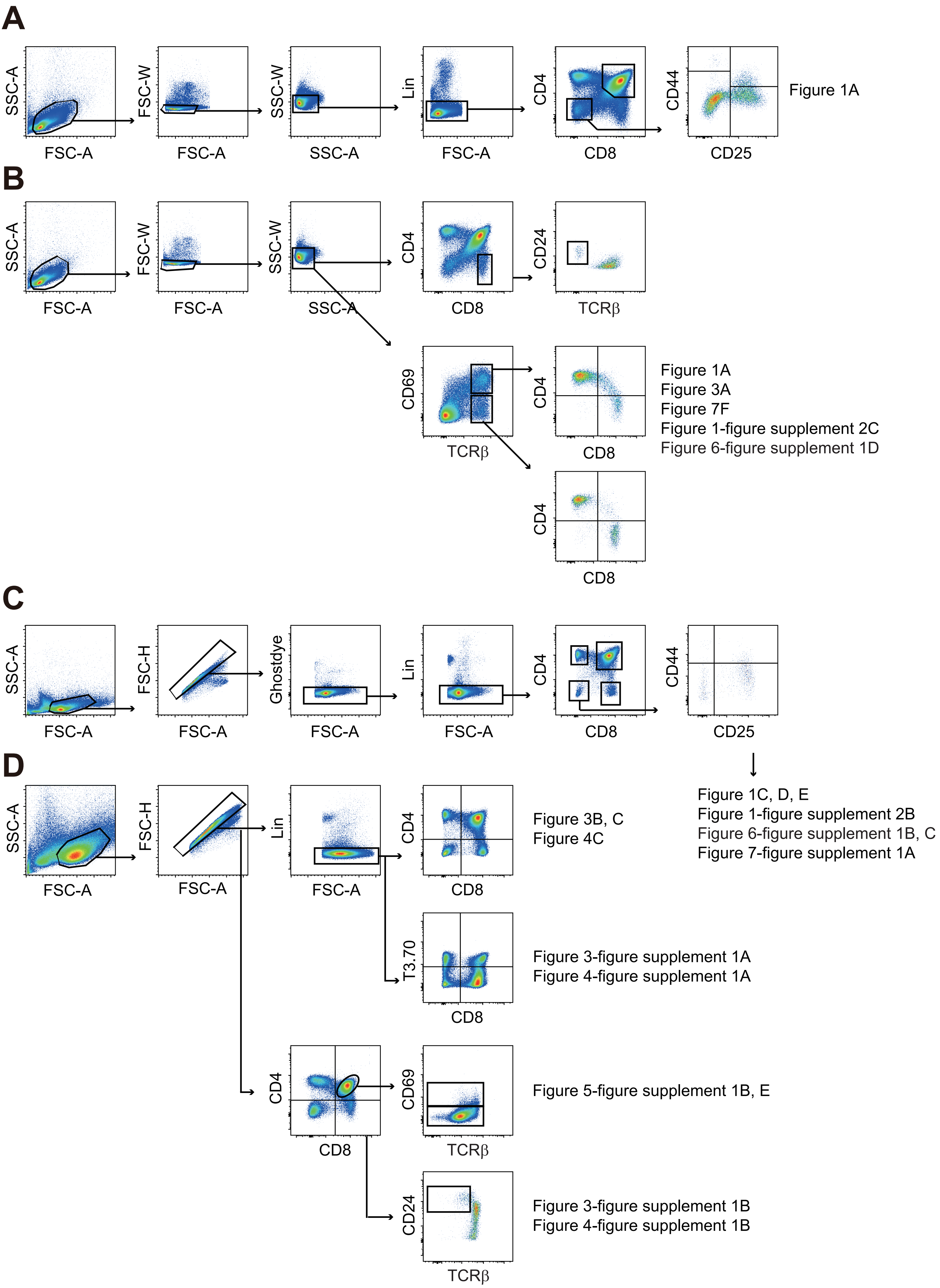

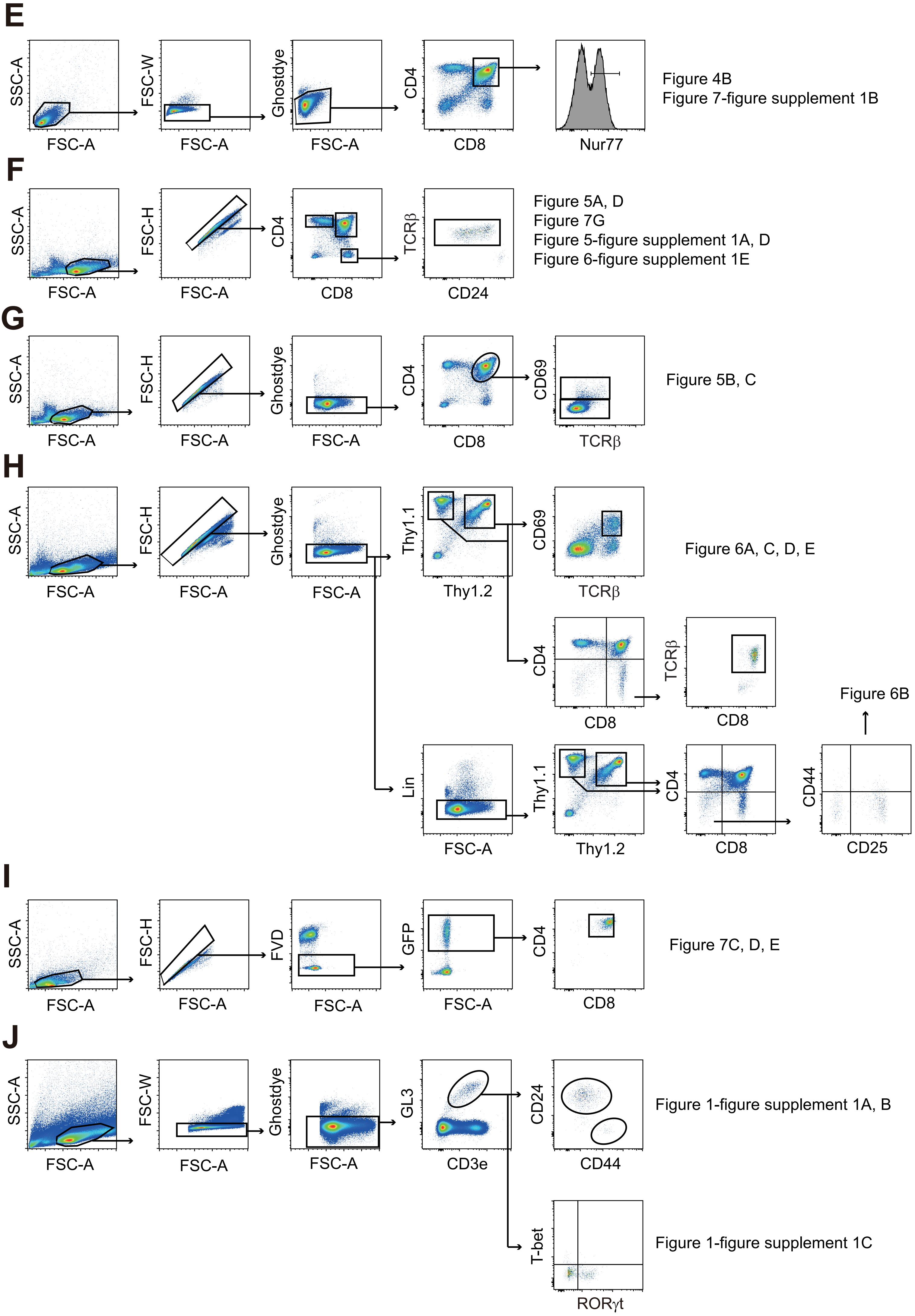
E
SSC-A
FSC-A
FSC-W
FSC-A
Ghostdye
FSC-A
CD4
CD8
Nur77
Figure 4B
Figure 7-figure supplement 1B
F
SSC-A
FSC-A
FSC-H
FSC-A
CD4
CD8
TCRβ
CD24
Figure 5A, D
Figure 7G
Figure 5-figure supplement 1A, D
Figure 6-figure supplement 1E
G
SSC-A
FSC-A
FSC-H
FSC-A
Ghostdye
FSC-A
CD4
CD8
CD69
TCRβ
Figure 5B, C
H
SSC-A
FSC-A
FSC-H
FSC-A
Ghostdye
FSC-A
Thy1.1
Thy1.2
CD69
TCRβ
Figure 6A, C, D, E
CD4
CD8
TCRβ
CD8
Figure 6B
Lin
FSC-A
Thy1.1
Thy1.2
CD4
CD8
CD44
CD25
I
SSC-A
FSC-A
FSC-H
FSC-A
FVD
FSC-A
GFP
FSC-A
CD4
CD8
Figure 7C, D, E
J
SSC-A
FSC-A
FSC-W
FSC-A
Ghostdye
FSC-A
GL3
CD3e
CD24
CD44
Figure 1-figure supplement 1A, B
T-bet
RORγt
Figure 1-figure supplement 1C

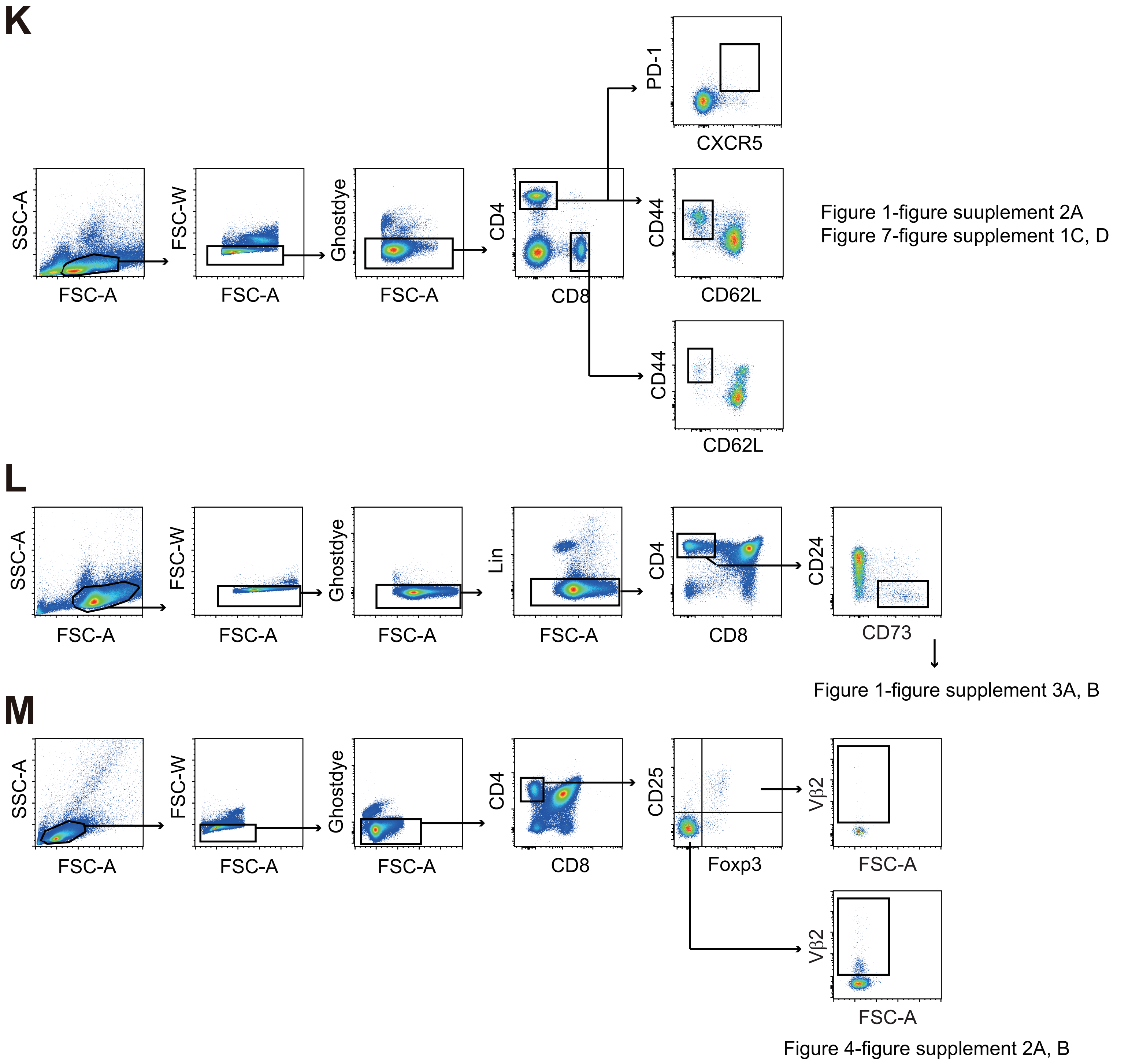

Supplementary file 4. Flow cytometry gating strategy.

(A) Sequential gating strategy for CD44- and CD25-expressing double-negative (DN) cells from *Cic*$^{FLAG/FLAG}$ mice.
(B) Sequential gating strategy used for CD24^{hi}TCRβ^{lo} immature single-positive (ISP) cells and T cells expressing CD69 and TCRβ.
(C) Sequential gating strategy for DP, CD4 SP, CD8 SP, and DN cells expressing CD44 and CD25.
(D) Sequential gating strategy for T cells from TCR transgenic mice.
(E) Sequential gating strategy for Nur77 expression analysis in double-positive (DP) cells.
(F) Sequential gating strategy for CD5 expression analysis in DP, CD4 SP, and CD8 SP (TCRβ^{hi}) cells.
(G) Sequential gating strategy for CD69^{+} and CD69^{-} DP cells.
(H) Sequential gating strategy for analysis of T cells from mixed bone marrow (BM) chimeric mice.
(I) Sequential gating strategy for GFP^{+} DP cells.
(J) Sequential gating strategy for γδT cells expressing CD24, CD44, T-bet, and RORγt.
(K) Sequential gating strategy for splenic T cells expressing CD44 and CD62L.
(L) Sequential gating strategy for CD73^{+}CD24^{lo}CD4^{+} T cells.
(M) Sequential gating strategy for CD4^{+} non-Treg and Treg Vβ2 chain. Other Vβ chains were gated with the same gating strategy.
